# The Interactive Role of Climatic Transfer Distance and Overstory Retention on Douglas‐Fir Seedling Survival and Height Growth in Interior British Columbia

**DOI:** 10.1111/gcb.70027

**Published:** 2025-01-24

**Authors:** Thomson C. Harris, W. Jean Roach, Erin M. Miller, Suzanne W. Simard

**Affiliations:** ^1^ Faculty of Forestry, Forest Sciences Centre The University of British Columbia Vancouver British Columbia Canada

**Keywords:** assisted migration, climate change, climatic transfer distance, forest harvesting, interior Douglas‐fir, overstory retention, regeneration, silviculture systems

## Abstract

The future climatic niche of interior Douglas‐fir ( *Pseudotsuga menziesii* var. *glauca* [Mirb.] Franco) is expected to have little spatial overlap with its current range due to climate change. The resulting misalignment of the climatic niche and species distribution is expected to result in many forests becoming maladapted in their current location, thus increasing vulnerability to disturbance and reducing productivity. This novel study examined the individual and interactive effects of climatic transfer distance and silviculture systems on planted 3‐year‐old Douglas‐fir seedlings across the natural range of interior Douglas‐fir in British Columbia. Several climatic transfer distance variables were considered, and the silviculture systems tested comprised the following gradients of tree retention: 0% retention (clearcut), 10% dispersed retention (seed‐tree), 30% aggregate retention, and 60% aggregate retention with thinning from below. Using linear mixed effect models, we found that survival and height were positively correlated with movements of seedlings to warmer, wetter, and more humid climates. Moisture availability had a stronger influence than temperature, indicating that seedlings transferred to warmer but more arid climates would experience decreased survival and height. Where seedlings were transferred to climates with greater frost frequency or decreased humidity, greater retention of overstory trees improved survival and height. Conversely, movements to more favorable climatic conditions (warmer and wetter) resulted in improved survival and height where overstory retention was low. Our findings suggest that genetic reshuffling of populations through assisted migration could benefit from overstory retention where stressful climatic conditions due to aridity or increased frost frequency occur.

## Graphical abstract

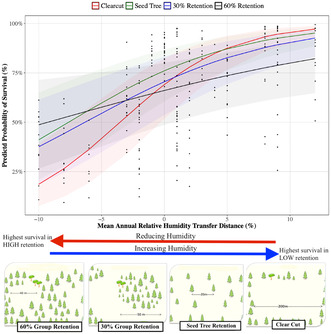

## 1 Introduction

Climate models predict long‐range shifts in the climatic niches of tree species and low spatial overlap between current and projected ecological zones (Hamann and Wang 2006). Interior Douglas‐fir ( *Pseudotsuga menziesii* var. *glauca* [Mirb.] Franco) is expected to become maladapted in its current distribution, which may reduce its productivity and increase its vulnerability to natural disturbances (Rehfeldt, Jaquish, Sáenz‐Romero, et al. 2014). The current distribution of interior Douglas‐fir extends from Mexico to central British Columbia (BC), Canada, where it is an ecologically and economically important species (Meidinger and Pojar 1991). BC's forests have been traditionally managed under the assumption of a stable climate (Spittlehouse and Stewart 2003). However, this assumption threatens juvenile trees, which have a low tolerance to climatic variation, thus increasing their vulnerability to mortality from maladaptation and creating a possible challenge for post‐harvest forest regeneration (Hamann and Wang 2006; Spittlehouse and Stewart 2003).

Large openings in forest canopies often create amplified surface temperatures that can increase the risk of mortality in the early establishment of seedlings (Stathers 1983). These openings can be created by clearcutting with reserves, which is the predominant silvicultural method in BC (Environmental Reporting BC 2021). Silvicultural systems that retain overstory trees, such as shelterwood, seed‐tree, and group selection, may offer better chances at regeneration than clearcutting (Franklin et al. 2002; Day, Koot, and Wiensczyk 2011; Bose, Nelson, and Olson 2020; Pommerening 2023). These systems sustain biological legacies that provide seed sources and moderate microclimate conditions to better protect seedlings (Langvall and Örlander 2001; Franklin et al. 2002; Heithecker and Halpern 2006; Rambo and North 2009; Baker et al. 2014; Pommerening 2023). Understanding how these silviculture systems will influence regeneration as the climate changes will be critical to managing interior Douglas‐fir ecosystems.

Seed selection for reforestation of harvested areas has conventionally been based on the concept that “local is best” (MacKenzie and Meidinger 2017; Mahony, MacKenzie, and Aitken 2018). Tree populations adapt to their local climate and thus are expected to perform better than non‐local seed sources (MacKenzie and Mahony 2021). However, climate change is transforming the forests of BC. The Biogeoclimatic Ecosystem Classification (BEC) zone boundaries have already shifted 23% since they were delineated in the 1970s, and novel climatic envelopes are expected to develop in the future (Wang et al. 2012; Mahony, MacKenzie, and Aitken 2018). Interior Douglas‐fir naturally populates south‐central BC within three BEC zones: the Interior Douglas‐fir (IDF), Interior Cedar Hemlock (ICH), and Sub‐boreal Spruce (SBS) zones (BECweb 2016). As the climate changes, these zones are expected to have hotter and drier summers paired with warmer and wetter winters, which may create previously undocumented climatic conditions (Mahony, MacKenzie, and Aitken 2018). Interior Douglas‐fir's climatic niche is projected to expand northward and westward (MacKenzie and Meidinger 2017), and with local climatic conditions changing, the “local is best” concept may no longer be applicable (Mahony, MacKenzie, and Aitken 2018). Seedlings may need to be sourced from non‐local provenances for successful reforestation (Mahony, MacKenzie, and Aitken 2018; Park and Rodgers 2023).

Genetic variability in response to a climatic gradient has been demonstrated among interior Douglas‐fir populations in provenance trials, indicating genetic reshuffling of natural populations is possible (Rehfeldt 1989). Interior Douglas‐fir shows a clinal response to winter temperatures (Rehfeldt, Leites, et al. 2014). By comparison, precipitation elicits a weaker clinal response from interior Douglas‐fir but plays a critical role in species distribution (Rehfeldt, Leites, et al. 2014). Within‐population assisted migration (AM) is being considered as a method for helping natural populations adapt to the changing climate more quickly than they could without assistance (Aitken and Whitlock 2013). Genetic reshuffling done via AM involves translocating individuals of a species to areas, often of either higher elevation or latitude, within the natural geographic range of that species (Aitken and Whitlock 2013; Charles and Stehlik 2021). AM experiments, done via provenance trials, are primarily studied in nurseries and clearcuts; AM is being applied cautiously in operational forest regeneration (Campbell 1979; Ying and Yanchuk 2006; Aitken and Whitlock 2013; Eilmann et al. 2013; Ye and Jayawickrama 2013; O'Neill, Stoehr, and Jaquish 2014; Rehfeldt, Leites, et al. 2014; Bansal et al. 2015; Neophytou et al. 2016; Pedlar, McKenney, and Lu 2021). Provenance trials have not been conducted across a range of silvicultural systems.

Silviculture systems that retain overstory trees are often designed to mimic natural disturbance regimes and assist natural regeneration (e.g., Malcolm, Mason, and Clarke 2001; Franklin et al. 2002; Day, Koot, and Wiensczyk 2011; Raymond et al. 2018; Lafleur, Harvey, and Mazerolle 2019; Bose, Nelson, and Olson 2020; Power et al. 2022). Historically, the most common natural disturbances in interior Douglas‐fir ecosystems have included low‐ to medium‐severity stand‐maintaining fires and small‐scale insect and pathogen mortality (Hessburg et al. 2019; Leclerc, Daniels, and Carroll 2021). In both cases, partial canopies and refugia of overstory trees often remain (Baker, Veblen, and Sherriff 2007; Agne et al. 2018).

A partial canopy of overstory trees reduces microclimatic variation (Heithecker and Halpern 2006) and extreme weather effects (Dai 2011; Day, Koot, and Wiensczyk 2011; Baldwin et al. 2019) by reducing temperature fluctuations, direct solar radiation, moisture loss, and frost (Langvall and Örlander 2001; Rambo and North 2009; Baker et al. 2014). This can favor regeneration of moderately shade‐tolerant interior Douglas‐fir (Williams, Messier, and Kneeshaw 1999). This is seen in moisture‐limited ecosystems, where retaining overstory trees can improve germination for interior Douglas‐fir by creating a more humid microclimate (LeMay, Pommerening, and Marshall 2009). By contrast, clearcutting aims to mimic stand‐replacing fires and to maximize growing space for regenerating stands (Curtis et al. 1998). This exposes seedlings to more arid conditions, which can diminish their regeneration success (Chen, Franklin, and Spies 1993). Clearcutting does not match the predominant natural disturbance regimes or ecophysiological requirements of interior Douglas‐fir (Franklin et al. 2002).

This novel field‐based provenance experiment examines the survival and height growth of interior Douglas‐fir seedlings in response to climatic transfer distance (CTD) under different silvicultural systems with varying levels of overstory retention. It is the first field experiment to our knowledge that investigates whether partial retention silviculture can be used to enhance the success of assisted migration as an adaptive approach for forest stewardship in a changing climate. The experiment was situated in BC, Canada, which represents the northern portion of interior Douglas‐fir's natural range. The objective was to determine the individual and interactive effects of CTD from provenance of origin and silvicultural system on survival and height growth of interior Douglas‐fir seedlings. Three hypotheses were tested: first, increasing CTD from the provenance of origin to wetter and warmer conditions would improve seedling survival and growth; second, increasing overstory tree retention would increase seedling survival but reduce height growth; third, there would be an interactive effect between CTD and overstory tree retention, where increasing overstory retention would improve survival but reduce growth for provenances moved to areas that are colder or more arid than their places of origin.

## 2 Methods

### 2.1 Study Area

**FIGURE 1:**
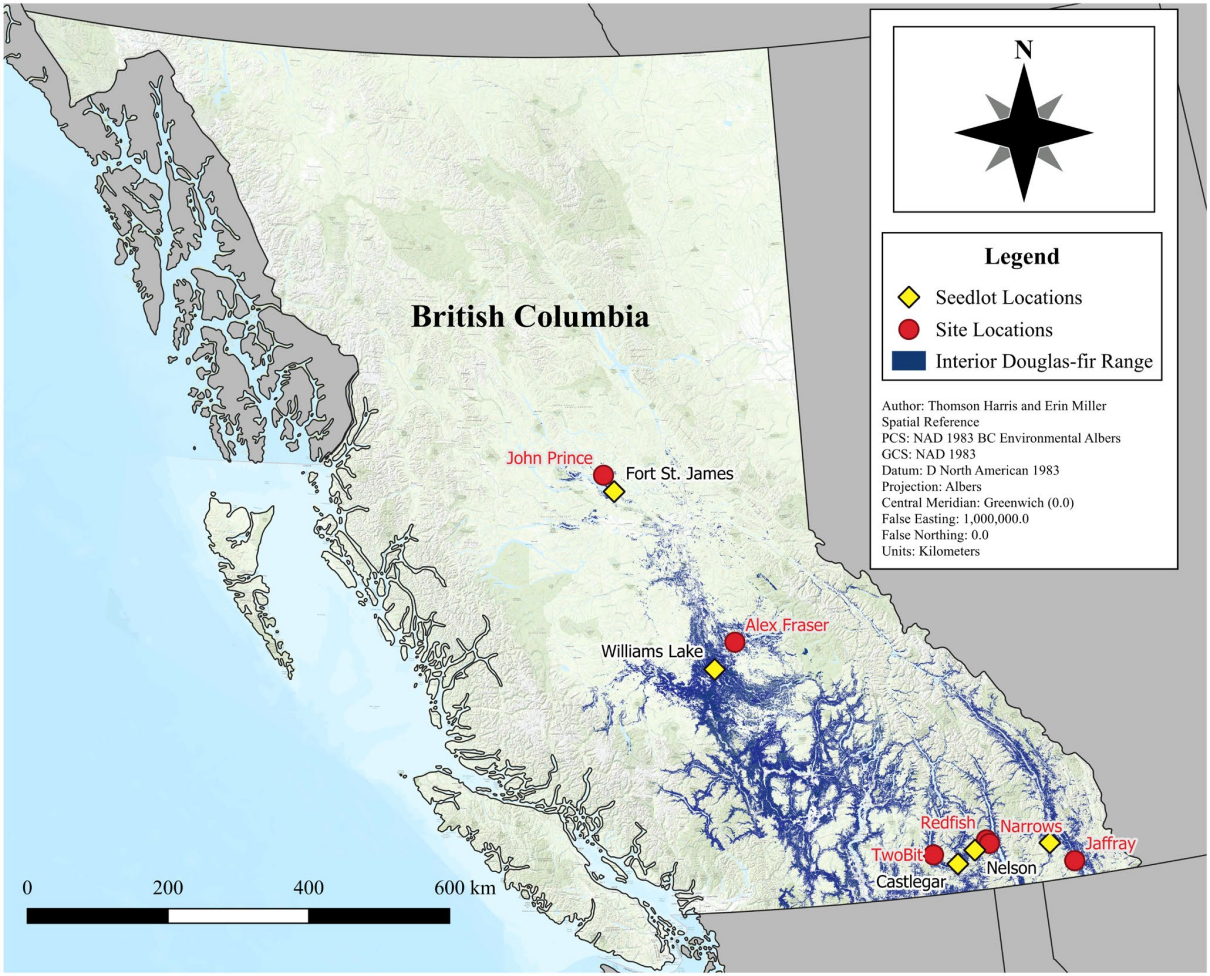
Map of study and seed lot locations showing the range of Interior Douglas‐fir in British Columbia. The map lines delineate study areas and do not necessarily depict accepted national boundaries.

**FIGURE 2:**
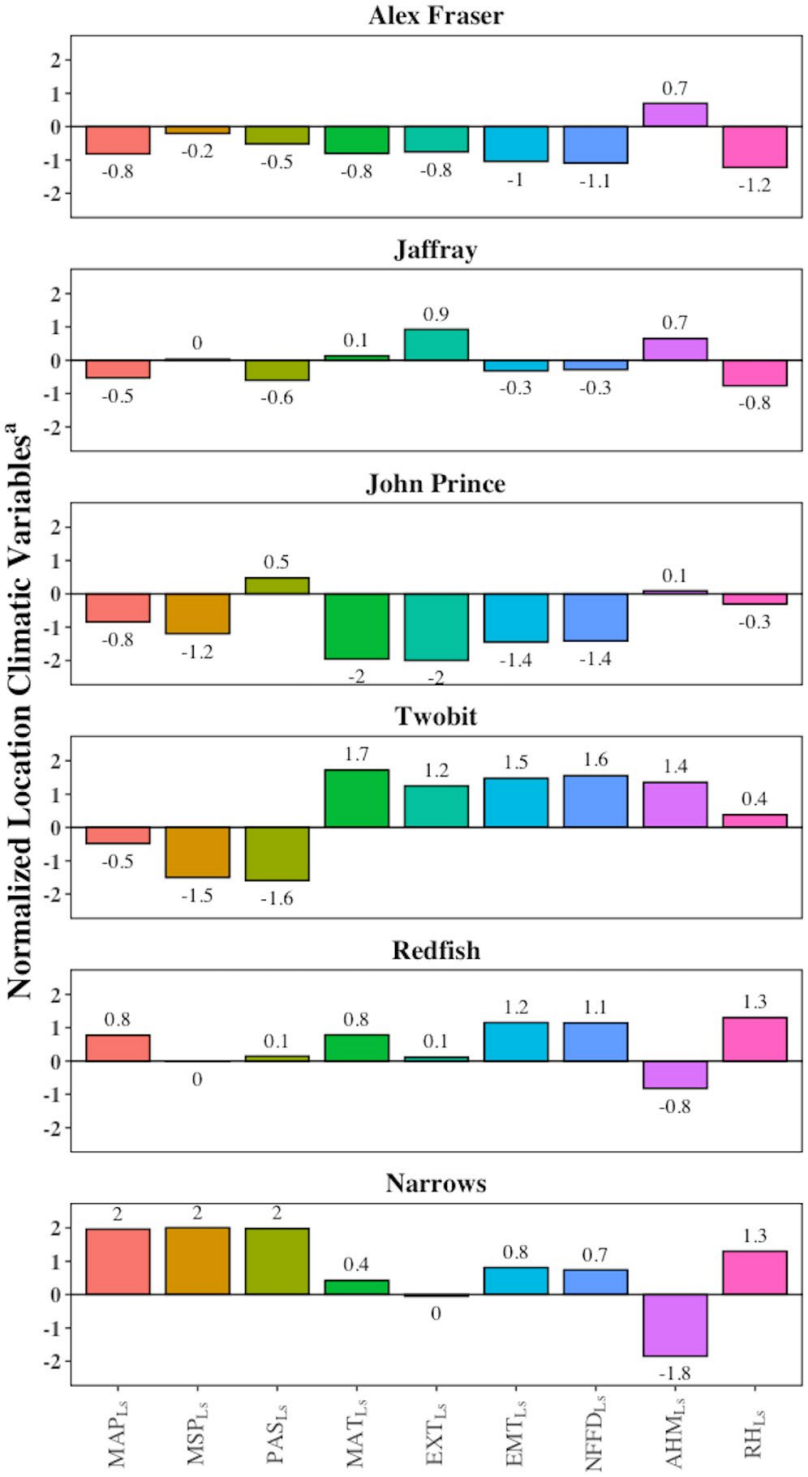
Summary of the climate at all locations. Locations are arranged by increasing relative humidity. Scaled location climatic variables are scaled relative to the other locations, where zero is the mean location climate across all sites. ^a^AHM_Ls_ = Scaled Location Annual Heat Moisture Index; EMT_Ls_ = Scaled Location Extreme Minimum Temperature (°C); EXT_Ls_ = Scaled Location Extreme Maximum Temperature (°C); MAP_Ls_ = Scaled Location Mean Annual Precipitation (mm); MAT_Ls_ = Scaled Location Mean Annual Temperature (°C); MSP_Ls_ = Scaled Location Mean Summer Precipitation (mm); NFFD_Ls_ = Scaled Location Number of Frost‐Free Days; PAS_Ls_ = Scaled Location Precipitation as Snow (mm); RH_Ls_ = Scaled Location Mean Annual Relative Humidity (%).

This study was conducted at six locations within the natural distribution of interior Douglas‐fir in British Columbia, Canada, from Cranbrook (49.27 N, 115.38 W) to Fort St. James (54.65 N, 124.43 W) (Figure 1). The study sites are located in the IDF, ICH, and SBS BEC zones and span a 900 km long climatic gradient with elevations ranging between 630 and 1250 m. Scaled location climates show site characteristics relative to the other sites within the study (Figure 2). John Prince, the most northern site, was the coldest; Two‐bit, the most southern, was the warmest. Narrows was the wettest site, and Two‐bit was the driest (Table 1). Forests at the six study locations were mature (82–123 years old) and originated from stand‐replacing wildfires. They were at least one‐third Douglas‐fir (by basal area) and, within individual locations, had a consistent species mix and stand structure. The sites had medium soil moisture and nutrient regimes as defined in the Land Management Handbook 25 (MFR and MoE 2010) and were predominantly south or west aspects and had gentle slopes.

**TABLE 1:** Characteristics of the study locations.

	Narrows	Redfish	John Prince	Jaffray	Alex Fraser	Two‐bit
Location details						
Logging date	Oct 2018	June 2017	Jan‐Mar 2018	June 2017	Nov 2018	Winter 2019/20
Planting date	2020	June 2018	June 2018	May 2018	2020	2020
Survey date	July 2021	June–July 2020	Aug 2020	Aug 2020	Sept‐Aug 2021	Aug 2021
No. of blocks	1	2	2	3	3	1
No. of plots	4	8	8	12	12	4
Geographic variables						
Nearby town	Nelson	Nelson	Fort St James	Cranbrook	Williams Lk	Castlegar
Latitude (°N)	49.58	49.63	54.65	49.21	52.45	49.52
Longitude (°W)	116.98	117.03	124.43	115.37	121.75	118.10
Elevation (m)	1080	890–940	870–920	1060–1100	930–965	600
Site variables						
Biogeoclimatic variant^a^	ICHdw1	ICHdw1	SBSdw3	IDFdm2	IDFdk3, ICHmk3, SBSdw1	ICHdw1
Site series	101/104	101/104	01	01	01	101/104
Climatic variables^b^						
MAP_L_ (mm)	1059	868	593	618	532	653
MSP_L_ (mm)	313	268	240	249	256	227
PAS_L_ (mm)	321	212	232	168	173	109
MAT_L_ (°C)	5.1	6.8	2.3	5.3	4.4	7.7
EXT_L_ (°C)	35.4	35.7	31.8	37.2	34.1	37.8
EMT_L_ (°C)	−28.8	−27.2	−39.4	−34.1	−37.5	−25.7
NFFD_L_	205	215	152	180	160	225
AHM_L_	14.3	19.4	20.8	24.7	27.3	27.2
RH_L_ (%)	71	71	64	62	60	67

^a^ ICHdw1 = West Kootenay Dry Warm Interior Cedar Hemlock; ICHmk3 = Horsefly Moist Cool Interior Cedar Hemlock; IDFdk3 = Fraser Dry Cool Interior Douglas‐fir; IDFdm2 = Kootenay Dry Mild Interior Douglas‐fir; SBSdw1 = Horsefly Dry Warm Sub‐Boreal Spruce; SBSdw3 = Stuart Dry Warm Sub‐Boreal Spruce (Lloyd et al. 1990; Braumandl and Curran 1992; DeLong, Tanner, and Jull 1993; Steen and Coupe 1997).

^b^ Climate data are 1981–2010 averages obtained from ClimateWNA v7.40 (Wang et al. 2012). AHM_L_ = Location Annual Heat Moisture Index; EMT_L_ = Location Extreme Minimum Temperature (°C); EXT_L_ = Location Extreme Maximum Temperature (°C); MAP_L_ = Location Mean Annual Precipitation (mm); MAT_L_ = Location Mean Annual Temperature (°C); MSP_L_ = Location Mean Summer Precipitation (mm); NFFD_L_ = Location Number of Frost‐Free Days; PAS_L_ = Location Precipitation as Snow (mm); RH_L_ = Location Mean Annual Relative Humidity (%).

### 2.2 Experimental Design and Treatments

**FIGURE 3:**
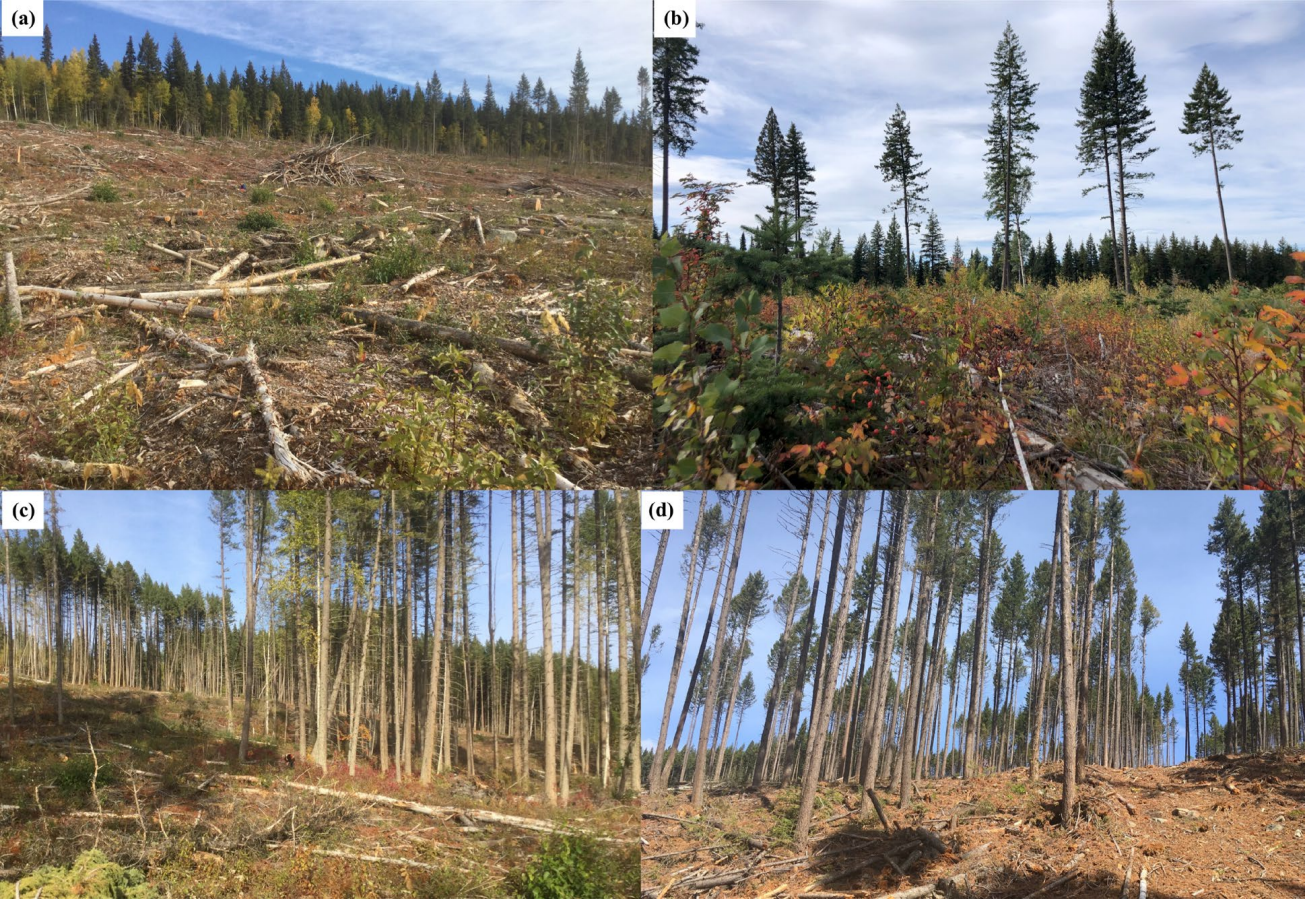
Images of silviculture systems used as treatments. (a) 0% retention (clear‐cut). (b) 10% dispersed retention (seed‐tree). (c) 30% aggregate retention. (d) 60% aggregate retention with thinning from below.

The study had a hierarchical split‐plot randomized block experimental design, where locations were selected to cover the range of climatic conditions for interior Douglas‐fir in BC (Figure 1). At each location, 4 ha treatment units (plots) were nested in up to three replicate blocks. The harvesting methods used for the treatment units were clear‐cut (0% retention); seed‐tree retention (10% retention; 25 mature trees ha^−1^ retained in a uniform dispersed distribution); 30% aggregate retention (30% of the land base unharvested in 30–40 m patches); and 60% aggregate retention (60% of the largest trees retained using thinning from below by reaching into the uncut areas with a machine; Figure 3; see also Figure S1 for treatment block layout). Selected harvesting methods were intended to mirror commonly used and studied commercial methods in BC (Curtis et al. 1998; Mitchell and Beese 2002; Day, Koot, and Wiensczyk 2011). Provenances were planted within the treatment units. The intersection of provenance within a specific treatment unit is referred to as a “split‐plot”.

Five provenances of interior Douglas‐fir were planted in a 1‐ha measurement plot centrally located in each of the 4 ha treatment units (Table 2). Provenances were planted at random with a spacing of between 7.5–10 m between seedlings of the same provenance, creating an overall spacing of 2.2–2.6 m (1400–1600 stems ha^−1^). B class seed, defined as seed collected from wild stands (BC Forest Service 1995), was used for all provenances. Planting followed the year after harvest, which occurred 2018–2020 (Table 1). Planted seedlings were tagged to identify the provenance of origin.

**TABLE 2:** Characteristics of the seedlots used.

Seedlot name (Number)	Nelson (19913)	Fort St. James (53614)	Cranbrook (53205)	Williams Lake (8491)	Castlegar (19905)
Geographic variables					
Latitude (°N)	49.62	54.43	49.64	52.46	49.78
Longitude (°W)	117.13	124.18	116.09	121.75	116.93
Elevation (m)	875	900	1050	900	610
Climatic variables^a^					
MAP_p_ (mm)	913	525	505	551	793
MSP_p_ (mm)	286	234	216	269	253
PAS_p_ (mm)	205	212	154	160	114
MAT_p_ (°C)	6.8	2.6	5.3	4.4	8.2
EXT_p_ (°C)	36.3	32.2	36.6	34.5	37.3
EMT_p_ (°C)	−26.6	−40.1	−35.6	−37.5	−23.7
NFFD_p_	218	151	181	162	241
AHM_p_	18.4	23.9	30.4	26.2	22.9
RH_p_ (%)	70	63	62	59	69

^a^ Climate data are 1981–2010 averages obtained from ClimateWNA v7.40 (Wang et al. 2012). AHM_p_ = Provenance Annual Heat Moisture Index; EMT_p_ = Provenance Extreme Minimum Temperature (°C); EXT_p_ = Provenance Extreme Maximum Temperature (°C); MAP_p_ = Provenance Mean Annual Precipitation (mm); MAT_p_ = Provenance Mean Annual Temperature (°C); MSP_p_ = Provenance Mean Summer Precipitation (mm); NFFD_p_ = Provenance Number of Frost‐Free Days; PAS_p_ = Provenance Precipitation as Snow (mm); RH_p_ = Provenance Mean Annual Relative Humidity (%).

Sampling occurred two growing seasons after planting at the split‐plot level. Seedlings were assessed in 3.99 m radius (0.005 ha) subplots located on a 10 × 10 m grid within the 1‐ha measurement plot. Seedlings were assessed as dead or alive, and the height of those alive was measured to the nearest 0.5 cm. Crown closure was visually assessed as a percentage between 0% and 100% in accordance with the Vegetation Resources Inventory—British Columbia Handbook (2016).

### 2.3 Data Analysis

Data analysis and visualization were done in R, version 4.4.1 (R Core Team 2024). Figures were created using the ggeffects and sjPlots packages (Lüdecke 2018, 2024). Both survival and height were analyzed using the lme4 package (Bates et al. 2015). The sample sizes for survival and height were *n* = 8040 and *n* = 5809, respectively. Survival was analyzed with a generalized linear mixed effect model (GLMM) using the *glmer* function with a binomial distribution and logarithmic link. Predicted probability of survival (PPS) was calculated using a logistics function. Height was analyzed with a linear mixed effects model using the *lmer* function. Height was transformed using the natural log to better fit model assumptions, which were assessed graphically. Outliers with irregularly negative residuals were dropped from the analysis if seedlings had been excessively damaged.

Nine climatic transfer distance variables were considered: mean annual precipitation transfer distance (MAP_td_, mm); mean summer precipitation transfer distance (MSP_td_, mm); precipitation as snow transfer distance (PAS_td_, mm); mean annual temperature transfer distance (MAT_td_, °C); extreme maximum temperature over 30 years transfer distance (EXT_td_, °C); extreme minimum temperature over 30 years transfer distance (EMT_td_, °C); number of frost‐free days transfer distance (NFFD_td_); annual heat‐moisture index transfer distance (AHM_td_, AHM_td_ = (MAT_td_ + 10)/(MAP_td_/1000)); and mean annual relative humidity transfer distance (RH_td_, %). Transfer distance was calculated as the climatic distance, in units of the climatic variable, from the provenance of origin to the location of planting in units on the climatic variable (e.g., MAP_td_ = location mean annual precipitation—provenance mean annual precipitation).

All climatic transfer distance variables were modeled as linear terms. It is common for climatic transfer distance to show a parabolic trend (Pedlar and McKenney 2017). However, this study is located at the northern end of Douglas‐fir's range, constraining the breadth of climatic transfer distances such that it was more fitting to model them as linear terms. To resolve scaling issues with both the *glmer* and *lmer* models, the climatic transfer variables were standardized around the mean and standard deviation using a *Z*‐score (Equation 1).

(1) $$Z=\frac{x_{i}-\text{Mean}}{\text{Standard Deviation}}$$

The remaining fixed effects were harvest method, harvest method‐climate interaction, percent crown closure, and crown closure‐climate interaction. The harvest method, crown closure, and their respective interaction terms were analyzed in separate models. Crown closure was square‐root transformed to better meet regression assumptions. Nested random effects were used to avoid pseudoreplication and to account for the spatial hierarchy within the experimental design. The random effects were location (the geographic area at which the treatments were done); block (the grouping of harvest treatments) within location; plot (the harvest treatment unit); and split‐plot (the provenance planted within a specific harvest treatment) (Equation 2). The full model is specified as:

(2) $$\begin{array}{cc} \text{Fixed Effects} & \text{Random Effects}\\ Y\sim A+B+A\times B+ & \text{Location}/\text{Block}/\text{Plot}/\text{Split}‐\text{plot} \end{array}$$

where *Y* = survival or height, *A* = a climatic transfer distance variable, *B* = harvest method or percent crown closure, and *A* × *B* = an interaction between individual effects *A* and *B*.

An exhaustive list of increasingly complex models was built for both survival and height. This was done by adding fixed effects to a null model. Using a likelihood ratio test (*lrtest*, lmtest package; Zeileis and Hothorn 2002), models of higher complexity were tested against simpler models of *n—*1 fixed effects to determine if the addition of a given variable was significant (Tables S1–S8).

Model significance was determined with an alpha level of 0.05. Goodness‐of‐fit was interpreted based on the models' AIC and conditional and marginal *R* ^2^ values. Where harvest method was significant, differences between harvest levels were computed using *emmeans* (from the emmeans package) with a Bonferroni adjustment (Lenth 2024). Where there was an interaction between harvest method and climatic transfer distance, the significant differences between the interaction slopes were computed using *emtrends* (Lenth 2024). For height, the Kenward‐Roger method was used to estimate the degrees of freedom. For survival, the degrees of freedom were estimated as asymptotic due to the constraints of the *glmer* function.

## 3 Results

**FIGURE 4:**
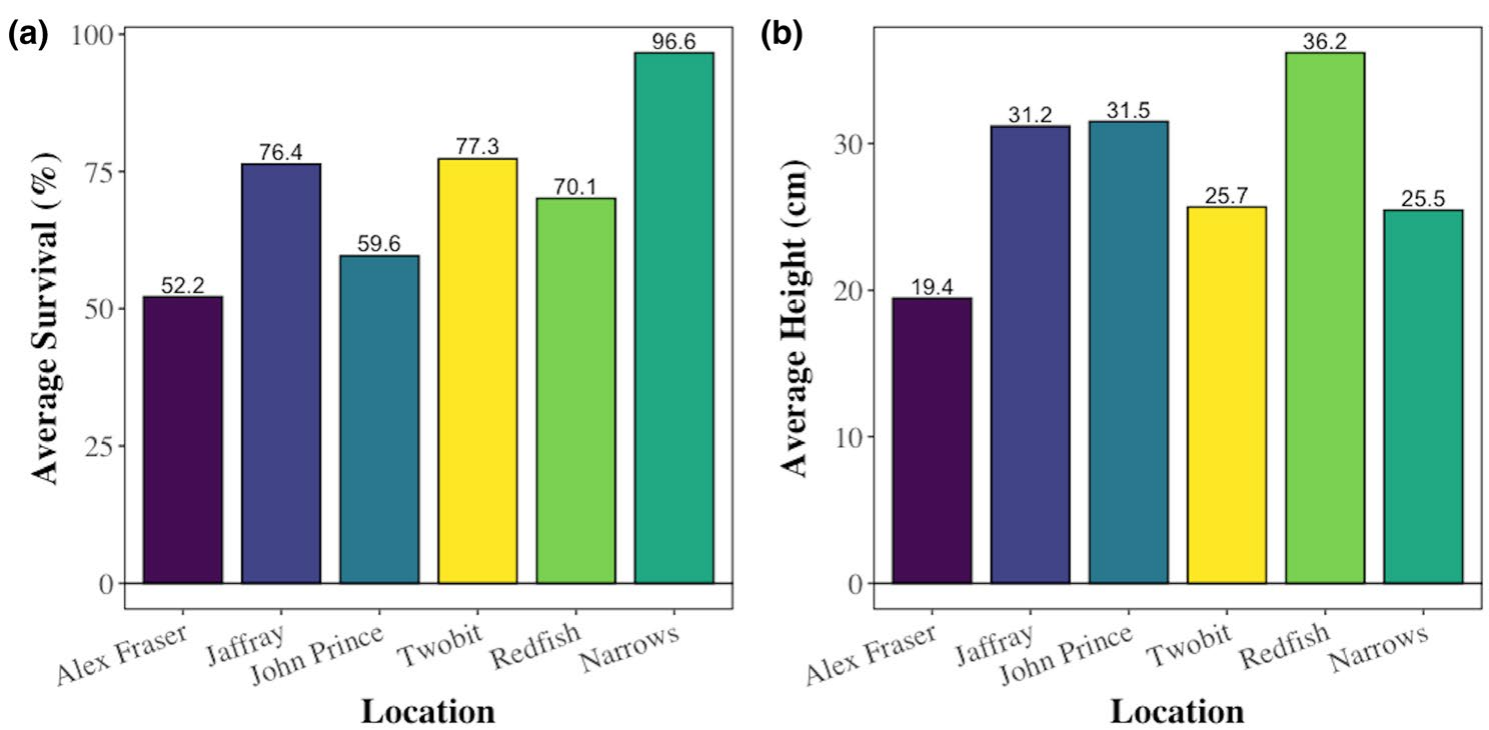
(a) Average survival (%) and (b) Average height (cm) at all locations. Locations are arranged by increasing relative humidity.

Average survival of 3‐year‐old planted seedlings across all harvesting methods ranged between 96.6% at Narrows and 52.2% at Alex Fraser (Figure 4a). The average survival of all seedlings irrespective of location was 72.3%. Average height ranged from 36.2 cm at Redfish to 19.4 cm at Alex Fraser (Figure 4b). The average height of all seedlings, irrespective of location, was 29.0 cm.

### 3.1 Climatic Transfer Distance

**FIGURE 5:**
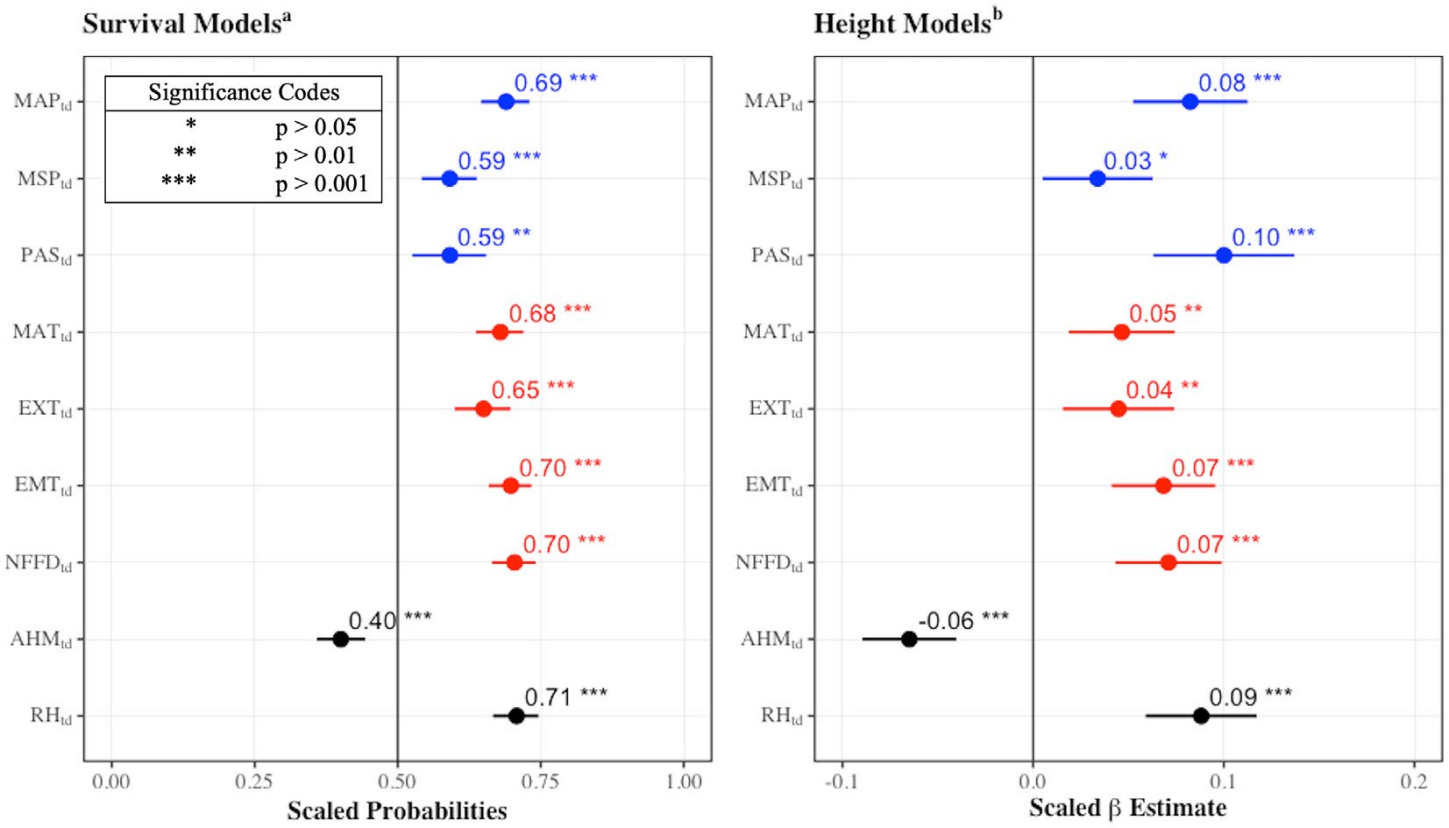
Scaled probabilities and scaled β coefficients of the effects of nine climatic transfer distance variables^c^ on predicted survival and height. Scaled probability indicates the change in probability of survival with a 1 unit change in the scaled variable. Similarly scaled β coefficients should be interpreted as the trend directionality, rather than slope directly. Asterisks indicate significance. Blue indicates precipitation related variables, red indicates temperature related variables, and black indicates moisture availability/aridity variables. ^a^survival ~ scaled climatic transfer distance*sqrt(crown closure) + (Location/Block/Plot/Split‐plot). ^b^ln(height) ~ scaled climatic transfer distance*sqrt(crown closure) + (Location/Block/Plot/Split‐plot). ^c^AHM_td_ = Annual Heat Moisture Index transfer distance; EMT_td_ = Extreme Minimum Temperature transfer distance (°C); EXT_td_ = Extreme Maximum Temperature transfer distance (°C); MAP_td_ = Mean Annual Precipitation transfer distance (mm); MAT_td_ = Mean Annual Temperature transfer distance (°C); MSP_td_ = Mean Summer Precipitation transfer distance (mm); NFFD_td_ = Number of Frost‐Free Days transfer distance; PAS_td_ = Precipitation as Snow transfer distance (mm); RH_td_ = Mean Annual Relative Humidity transfer distance (%).

Climatic transfer distance variables, MAP_td_, MSP_td_, PAS_td_, MAT_td_, EXT_td_, EMT_td_, NFFD_td_, and RH_td_, all had significant positive effects on survival with scaled probabilities ranging from 0.59–0.71. The aridity index, AHM_td_, had a significant negative effect on survival with a scaled probability of 0.40 (Figure 5). Scaled probability indicates the change in probability of survival associated with a 1‐unit change in scaled climatic transfer distance. Therefore, for AHM_td_, survival declined by 41% with a 1‐unit increase in scaled AHM_td_ and not an absolute change in AHM_td_. These values can be considered as the relative strength of the climatic transfer distance variable. Similarly, MAP_td_, MSP_td_, PAS_td_, MAT_td_, EXT_td_, EMT_td_, NFFD_td_, and RH_td_ all had significant positive associations with height, with scaled *β* coefficients ranging from 0.03 to 0.10. AHM_td_ had a significant negative association with height, with scaled *β* = −0.06. Similar to the scaled probabilities, the scaled *β* coefficients can be interpreted as the relative strength of the climatic transfer distances on height. These values for both survival and height were derived from the full models with crown closure (Equation 2).

**FIGURE 6:**
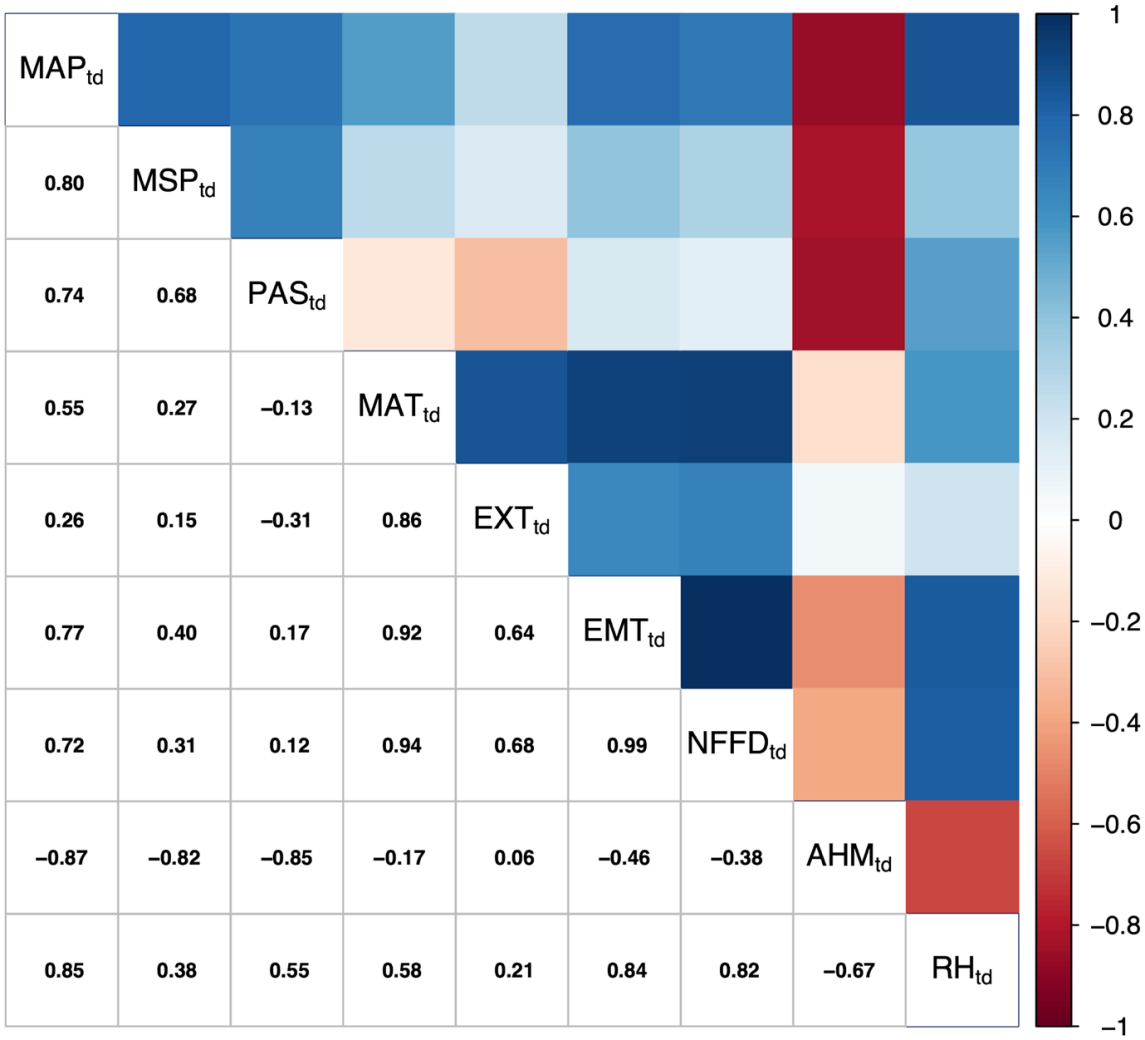
Correlation matrix between climatic transfer distance variables^a^. Positive correlation is indicated by positive values (blue) where 1 is perfect positive correlation. Negative correlation is indicated by negative values (red) where −1 is perfect negative correlation. Lightly shaded colours indicated weaker correlations where 0 is no correlation. ^a^AHM_td_ = Annual Heat Moisture Index transfer distance; EMT_td_ = Extreme Minimum Temperature transfer distance (°C); EXT_td_ = Extreme Maximum Temperature transfer distance (°C); MAP_td_ = Mean Annual Precipitation transfer distance (mm); MAT_td_ = Mean Annual Temperature transfer distance (°C); MSP_td_ = Mean Summer Precipitation transfer distance (mm); NFFD_td_ = Number of Frost‐Free Days transfer distance; PAS_td_ = Precipitation as Snow transfer distance (mm); RH_td_ = Mean Annual Relative Humidity transfer distance (%).

There were strong correlations between some of the CTD variables (Figure 6). The precipitation variables (MAP_td_, MSP_td_, and PAS_td_) were all positively correlated with each other. Similarly, the temperature variables (MAT_td_, EXT_td_, EMT_td_, and NFFD_td_) were all positively correlated. AHM_td_ was negatively correlated with all precipitation variables, while RH_td_ was positively correlated. Importantly, there was some degree of variation between almost all variables, meaning they explain different portions of the data.

### 3.2 Harvest Method Models

**FIGURE 7:**
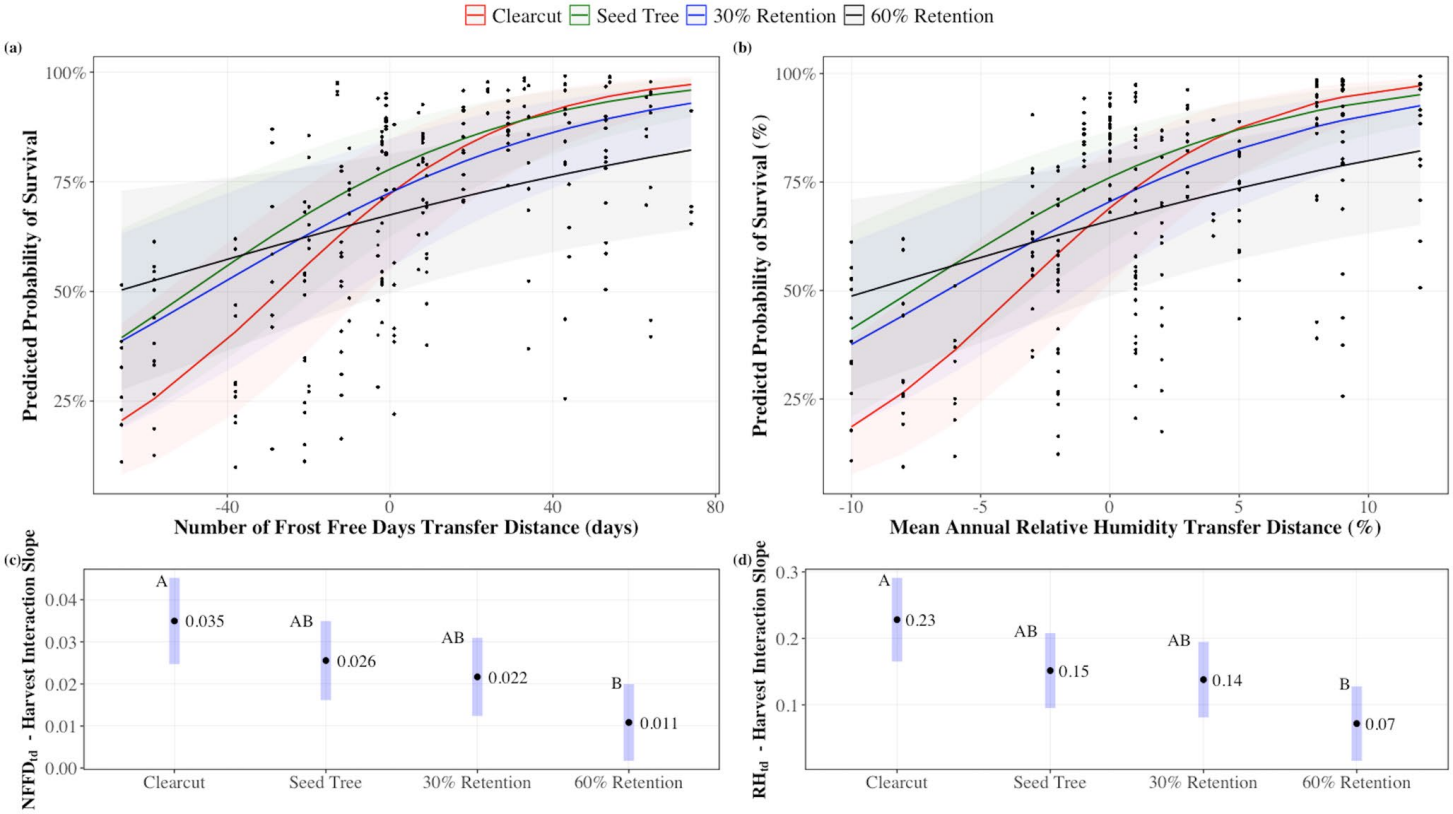
(a, b) The relationship between estimated probability of survival^a^ (%) and mean annual number of frost‐free days transfer distance (NFFD_td_, days) (Marginal and Conditional *R* ^2^ were 0.119 and 0.413, respectively) and mean annual relative humidity transfer distance (RH_td_, %) (Marginal and Conditional *R* ^2^ were 0.137 and 0.413, respectively) for each of the treatment types. (c, d) Estimated marginal slopes NFFD_td_ and RH_td_ for each treatment type. Different letters indicate significant differences. The clearcut and 60% retention treatment interaction slopes are significantly different for both the NFFD_td_ and RH_td_ models with *p* = 0.002 and *p* = 0.0009, respectively. ^a^Survival ~ Transfer Distance + Harvest + Transfer Distance*Harvest + (Location/Block/Plot/Split‐plot).

Harvest method did not significantly affect survival; however, survival was affected by significant interactions between harvest method and NFFD_td_, RH_td_ or EMT_td_ (Figure 7, Figure S2) (*p* = 0.0064, marginal *R* ^2^ = 0.119, conditional *R* ^2^ = 0.413 for NFFD_td_; *p* = 0.0040, marginal *R* ^2^ = 0.137, conditional *R* ^2^ = 0.413 for RH_td_). Predicted probability of survival (PPS) increased with NFFD_td_ and RH_td_ for all harvest methods (Figure 7a,b). With warmer and wetter conditions (increasing NFFD_td_ and RH_td_), lower retention treatments were more favorable for survival than the 60% retention. At a RH_td_ of +12% away from the provenance of origin (most positive movement), the PPS was 97% for the clearcut, 95% for the seed tree, 92% for the 30% retention, and 82% for the 60% retention treatment. Similar values occurred for increased NFFD_td_ movements. By contrast, the predicted probability of survival was better in the 60% retention treatment in the coldest and driest conditions. With a −10% movement in relative humidity from the provenance of origin (most negative movement), the PPS was 19% for the clearcut, 41% for the seed tree, 38% for the 30% retention, and 49% for the 60% retention treatment. The retention treatments had smaller effects on survival where the transfer distance was around 0, with PPS for the RH_td_ model at 69%, 76%, 70%, and 66% for the clearcut, seed‐tree, 30% retention, and 60% retention treatments, respectively (values similar for the NFFD_td_ model).

The estimated marginal (EM) slopes for each harvest method‐climatic transfer distance interaction indicate the difference in interaction between the climatic variable and harvest method. The interaction slope adjustments are untransformed from the generalized linear mixed effects model and do not directly represent a change in PPS. The EM slopes ranged from 0.011–0.035 and 0.07–0.23 for the NFFD_td_ and RH_td_ models, respectively, and were significantly different only between the clearcut and 60% retention treatment (Figure 7c,d).

**FIGURE 8:**
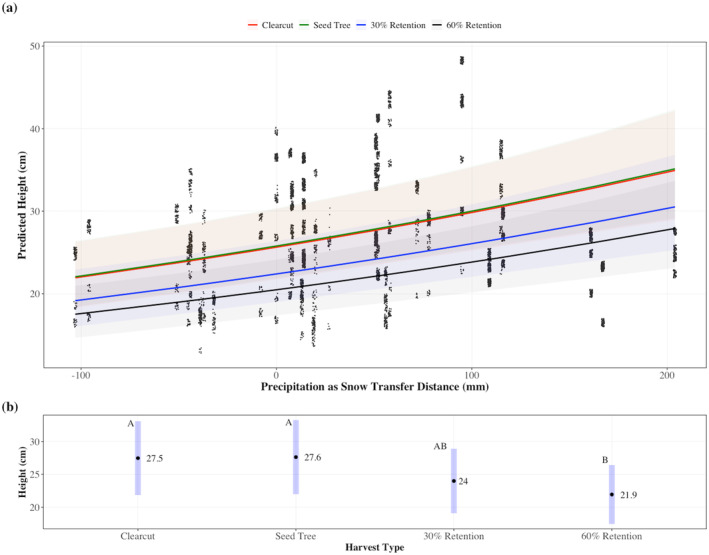
(a) The relationship between predicted seeding height^a^ (cm) and mean annual precipitation as snow transfer distance (PAS_td_, mm) for each treatment type. (b) Estimated marginal means for PAS_td_ for each treatment type. Different letters indicate significant differences. (Pairwise test, clearcut—60% retention *p*‐value = 0.016; Seed‐tree—60% retention *p*‐value = 0.012). Marginal and conditional *R* ^2^ were 0.095 and 0.388, respectively. 1 ln(height) ~ Transfer Distance + Harvest + Transfer Distance*Harvest + (Location/Block/Plot/Split‐plot).

Of all the CTD variables, PAS_td_ had the strongest effect on predicted height with a scaled *β* = 0.10 (marginal *R* ^2^ = 0.095, conditional *R* ^2^ = 0.388 for the PAS_td_ model) (Figures 5 and 8), but it did not interact with harvest method. The harvest method had a significant individual effect on height. There was no significant interaction between harvest method and any CTD variable for height. Height was significantly lower in the 60% retention treatment than the clearcut (*p* = 0.016) and seed‐tree treatments (*p* = 0.012, EM means of 21.9 cm vs. 27.5 cm or 27.6 cm, respectively) (Figure 8b). This trend in height was consistent across all climatic transfer variables modeled (Figures S3–S8).

### 3.3 Crown Closure Models

For both survival and height, the crown closure models performed better than the harvest method models. They consistently had a higher log‐likelihood and marginal *R* ^2^ and a lower AIC than their harvest method counterparts. The difference between model performance was greater for the height models than the survival models (Tables S1–S8).

**FIGURE 9:**
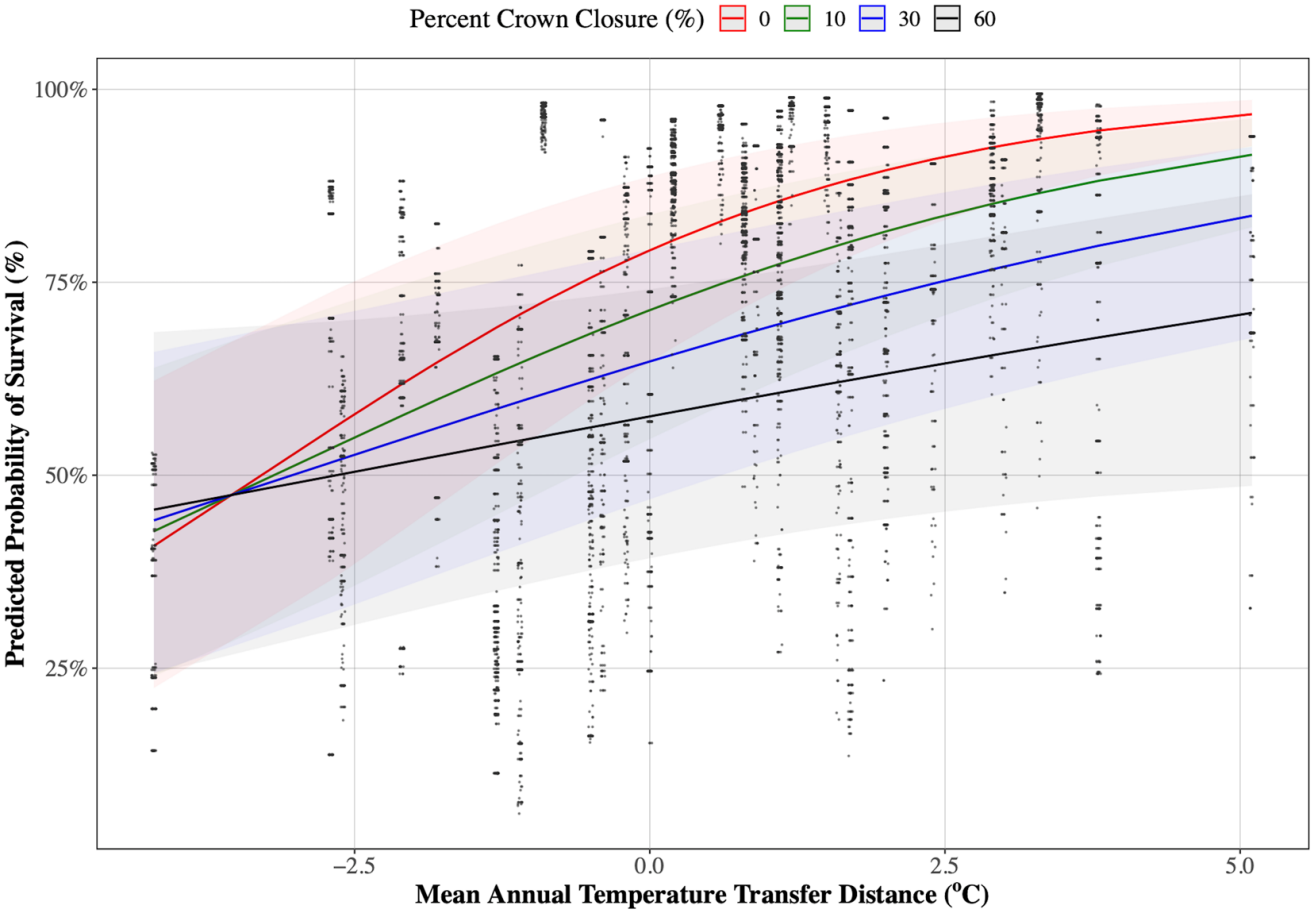
The relationship between predicted probability of survival^a^ (%) and mean annual temperature transfer distance (MAT_td_, °C) for different levels of crown closure (%). Marginal and conditional *R* ^2^ were 0.098 and 0.427 respectively. ^a^Survival ~ Transfer Distance + Crown Closure + Transfer Distance*Crown Closure + (Location/Block/Plot/Split‐plot).

Predicted probability of survival was negatively correlated with crown closure. There was also an interaction between crown closure and MAT_td_ (*p* = 1.078e‐6; marginal *R* ^2^ = 0.098; conditional *R* ^2^ = 0.427), EMT_td_, EXT_td_, MAP_td_, NFFD_td_, and RH_td_ (Figure 9, Figures S9–S13). At a MAT_td_ of −4.2°C away from the provenance of origin, there was a negligible difference in PPS between 0% and 60% crown closure. However, as MAT_td_ increased, PPS was greater with lower crown closure. PPS increased from 71% at 60% crown closure to 97% at 0% crown closure for a MAT_td_ of +5.1°C away from the provenance of origin. Similar trends occurred for EMT_td_, EXT_td_, MAP_td_, NFFD_td_, and RH_td_ (Figures S9–S13).

**FIGURE 10:**
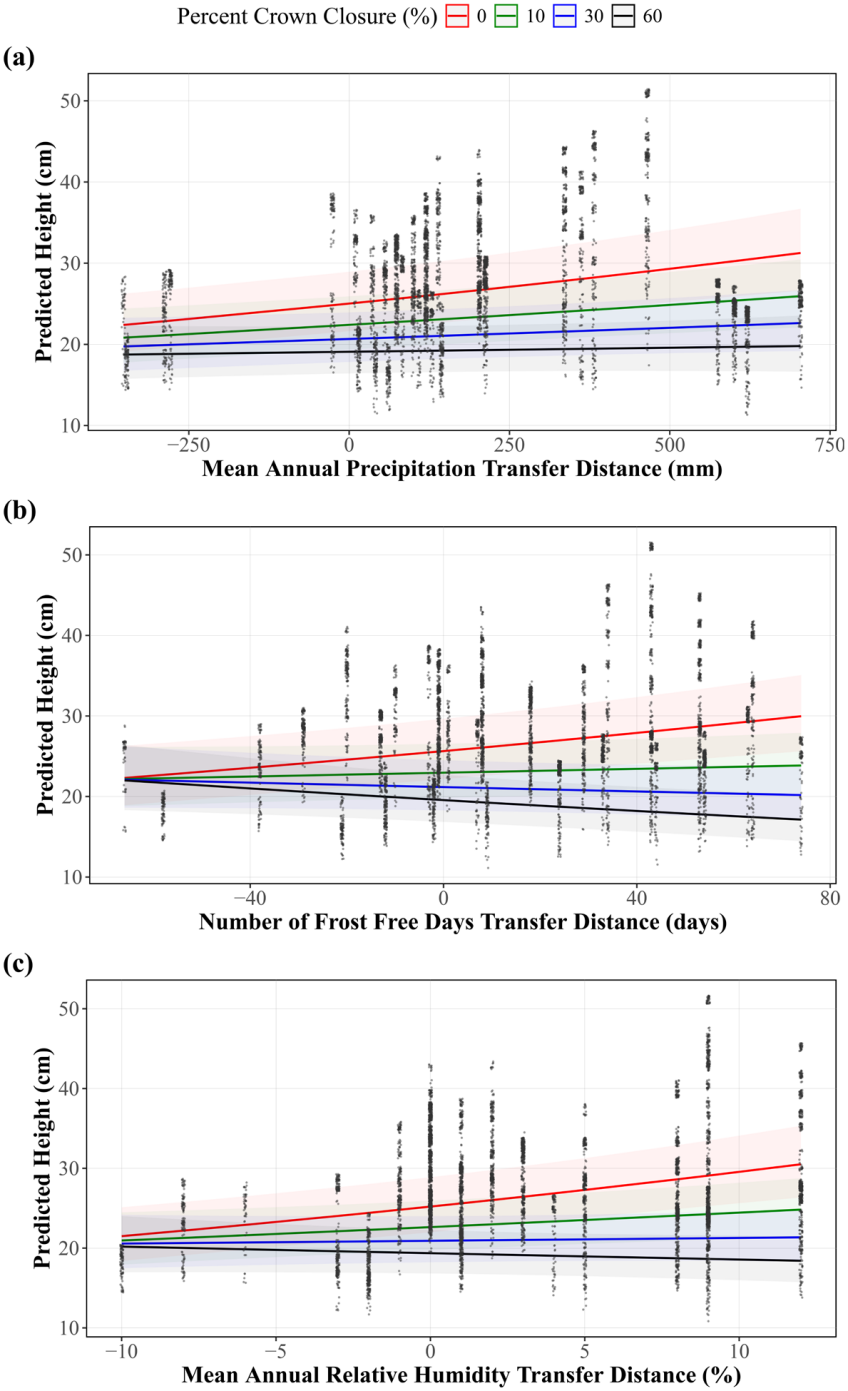
The relationship between predicted seedling height^a^ (cm) and (a) Mean annual precipitation transfer distance (MAP_td_, mm), (b) Mean annual number of frost‐free days transfer distance (NFFD_td_, days), and (c) Mean annual relative humidity transfer distance (RH_td_, %) for different levels of crown closure. Marginal and conditional *R* ^2^ were 0.074 and 0.382 for MAP_td_, 0.053 and 0.382 for NFFD_td_, and 0.102 and 0.407 for RH_td_ respectively. ^a^ln(height) ~ Transfer Distance + Crown Closure + Transfer Distance*Crown Closure + (Location/Block/Plot/Split‐plot).

Similar to survival, height was negatively related to crown closure (Figure 10a–c). The interactions between crown closure and MAP_td_, NFFD_td_, or RH_td_ were significant (*p* = 6.83e‐4, *p* = 5.85e‐10, and *p* = 2.94e‐8, respectively; marginal *R* ^2^ = 0.194, 0.371, and 0.200; conditional *R* ^2^ = 0.375, 0.198, and 0.367, respectively), as well as EMT_td_, EXT_td_, and MAT_td_ (Figures S14–S16). As MAP_td_, NFFD_td_, and RH_td_ increased, crown closure had a larger negative impact on height (Figure 9). For a transfer distance of +74 frost‐free days (the largest positive movement), predicted height was 30 cm at 0% crown closure while it was 17 cm at 60% crown closure. Conversely, in drier and colder climates (decreasing MAP_td_, NFFD_td_, and RH_td_), crown closure had little to no impact on seedling height. At an NFFD_td_ of −66 frost‐free days (the largest negative movement), the predicted height was 22 cm at 0%, 10%, 30%, and 60% crown closure. Similar values occurred across both MAP_td_ and RH_td_ (Figure 10a,c).

## 4 Discussion

### 4.1 Climatic Transfer Distance

The impacts of climate change in forests can be approximated by observing planted seedling response to climatic transfer distance (Pedlar and McKenney 2017; Leites and Benito Garzón 2023). We found that interior Douglas‐fir survival and height increased with movements to wetter climates (increasing MAP_td_, MSP_td_, and PAS_td_) across all harvest method treatments. This supports our first hypothesis that seedling performance will improve where transfers from stressful to more favorable climatic conditions occur. This finding is consistent with studies showing that the distribution and growth of interior Douglas‐fir are associated with precipitation and snowfall (Griesbauer and Green 2010; Griesbauer, Green, and O'Neill 2011; Rehfeldt, Jaquish, López‐Upton, et al. 2014). PAS_td_ had a particularly strong positive effect on height, suggesting that the snowpack plays a critical role in interior Douglas‐fir growth, whereas MAP_td_ had the strongest influence on survival. RH_td_ was also positively correlated with both survival and height, which is consistent with studies showing atmospheric drought negatively impacts interior Douglas‐fir growth (Jansen et al. 2014). Transferring trees to more arid climates (increasing AHM_td_) was negatively correlated with both survival and height, supporting Rehfeldt, Jaquish, López‐Upton, et al. (2014). The biogeoclimatic zones that interior Douglas‐fir inhabit are expected to become warmer with wetter winters in the future, which will likely result in a reduced snowpack (Mahony, MacKenzie, and Aitken 2018). This change in winter climate is expected to accompany hotter and drier summers and decreased annual precipitation (Mahony, MacKenzie, and Aitken 2018). Our findings support the idea that interior Douglas‐fir may become maladapted to its current distribution, as the expected decrease in annual precipitation is akin to the negative movement along the MAP_td_ gradient.

Interior Douglas‐fir survival and height increased with both mean annual and extreme maximum temperature transfer distance (MAT_td_ and EXT_td_) across all of the harvesting methods. This is similar to Peng et al. (2011), who found increased temperatures have been associated with increased growth in other northern coniferous species. However, when this is paired with increased drought, there is an expected increase in mortality. Our findings contrast with Case and Peterson (2005) and Chen and Hamann (2010), both of which found negative associations with high summer temperatures in certain conditions. This discrepancy is likely due to differences in study locations, where our experiment was situated only at the northern extent of interior Douglas‐fir's natural distribution.

In our study, in the northern portion of interior Douglas‐fir's extent, CTDs towards increased mean annual and extreme maximum temperature are associated with movements away from extreme cold, rather than movements away from moderate to very hot temperatures relative to interior Douglas‐fir's range of tolerance. As such we expect cold to be a stronger limiting growth factor than heat. The number of frost‐free days and extreme minimum temperatures transfer distance (NFFD_td_ and EMT_td_) had a stronger positive correlation with survival and height than MAT_td_ and EXT_td_. Douglas‐fir shows clinal relations with winter temperature, so it is not surprising to see a stronger relationship with these CTD variables (Rehfeldt, Leites, et al. 2014). NFFD_td_ and EMT_td_ are highly correlated variables but are not strongly correlated with snowfall (PAS_td_), which parallels the same trend in seedling survival and height growth.

It is important to note that the CTD variables were modeled as linear terms due to a relatively small sample size of provenances and resulting CTDs. Our results can only be applied to short CTDs and may not apply to larger movements. Survival and height growth generally show a parabolic trend in response to CTD. However, due to the location of this study, the breadth of CTD variables shows a linear portion of what is likely a larger parabolic trend (Leites et al. 2012).

### 4.2 Overstory Tree Retention

Overstory tree retention is comprised of the spatial pattern of the canopy and the extent of crown closure (Halpern et al. 2012). This is represented by harvest method (stand‐level treatment) and crown closure (percent cover experienced by individual seedlings), both of which affected seedling performance, partially supporting our second hypothesis. The harvest method interacted with CTD to affect survival, while crown closure had an interactive effect with CTD that affected both survival and height. This partially supports our third hypothesis.

The harvest method interacted with RH_td_ and NFFD_td_ to affect survival in complex ways. Seedlings moved to climates with increased aridity or frost frequency survived better in the 60% retention, 30% retention, and seed‐tree treatments compared to the clearcut treatment. Whereas seedlings moved to more favorable growing climates (increased humidity and increased number of frost‐free days) had better survival in the clearcut, seed‐tree, and 30% retention compared to the 60% retention treatment. Increased survival at higher retention levels in harsher climate conditions was likely due to the ability of overstory trees to reduce moisture loss and frost damage (Stathers 1983; Chen, Franklin, and Spies 1993; Heithecker and Halpern 2006; Rambo and North 2009). Seedlings transferred to wetter, warmer climates, by contrast, likely benefited from the greater light availability in the lower retention treatments (Chen, Klinka, and Kayahara 1996; Williams, Messier, and Kneeshaw 1999). Seedlings in the seed‐tree and 30% retention treatments performed well in both more favorable and harsher growing conditions. Moreover, survival for locally planted seedlings (CTD = 0) was effectively the same across all harvest methods.

By contrast to survival, seedlings grew taller in the clearcut and seed tree than in the 60% retention treatment regardless of climatic transfer distance. However, height growth was the same between the 30% retention, seed‐tree, and clearcut treatments, supporting studies showing interior Douglas‐fir can grow well in partial shade (Williams, Messier, and Kneeshaw 1999; Bose, Nelson, and Olson 2020).

Crown closure affected survival and height, but the extent of the impact depended on CTD. Where seedlings were moved to harsher growing conditions (decreasing MAT_td_), PPS was the same regardless of crown closure, but when moved to more favorable growing conditions (increased MAT_td_), PPS was greater at low crown closure levels. Similarly, height growth increased with movements to more favorable climates (i.e., increased MAP_td_, NFFD_td_, and RH_td_) where crown closure was low. This aligns with studies that found that light availability has a direct impact on height, as larger gaps in the canopy allow for direct sunlight (Chen, Klinka, and Kayahara 1996; Chen 1997; York, Battles, and Heald 2003; Vyse et al. 2006). However, predicted height in harsher climates (decreased MAP_td_, NFFD_td_, and RH_td_) was approximately the same for all values of crown closure. Similar to survival, this was likely due to the protective effects of overstory cover against moisture loss and frost (Stathers 1983; Chen, Franklin, and Spies 1993; Heithecker and Halpern 2006; Rambo and North 2009).

Crown closure explained more variation than harvest method did for both survival and height. However, the discrepancy is larger between height models than the survival models. The differences between the effects of harvest method and crown closure on survival and height growth are in part a result of the spatial distribution of retained trees. Average height growth was similar between the lower retention treatments when examined at the stand level, but more nuanced differences were evident with crown closure. Crown closure in the 30% and 60% retention treatments was not homogenous because the stands were harvested in aggregate patches, leaving variably sized canopy gaps. The harvest method looks at the broad stand‐level effects, while crown closure is a finer‐comb view of the immediate neighborhood of a seedling, thus explaining more variation. Height did not differ between the 30% retention and the clearcut or seed‐tree harvest methods (Figure 8), whereas predicted height substantially decreased as percent crown closure increased from 0% to 30% in good growing conditions (Figure 10). However, the effects of harvest method and crown closure would likely be more similar in methods that leave more evenly distributed overstory trees.

### 4.3 Forest Management Implications for a Changing Climate

Climate change is shifting forest ecosystems in British Columbia such that their historical BEC zones are increasingly incongruent with current delineations, and novel climatic envelopes are emerging. As a result, management plans created using current BEC boundaries are starting to lead to local regeneration failures (Mather et al. 2010; Wang et al. 2012; MacKenzie and Meidinger 2017; Mahony, MacKenzie, and Aitken 2018). Using assisted migration to aid adaptation is presented as an option to address this issue (Aitken and Whitlock 2013). Careful selection of silvicultural systems may help to minimize the risks and improve the survival of planting seedlings that are adapted to the projected climatic conditions of an area. The impact of increasing drought or frost, from a changing climate, on local seed sources can be considered akin to moving provenances to drier or colder areas. In these instances, seedlings benefit from increased overstory retention.

Considering the limiting factors on seedling growth is important when moving seedlings to new locations. In climatic situations where light availability is the limiting growth factor, we found that lower overstory retention was favorable to seedling survival and height growth. However, where moisture availability or frost was limiting, either due to regional climatic conditions, climate change, or assisted migration, we found that retaining higher cover of overstory trees helped seedling establishment by mitigating exposure to harsher microclimates. This aligns with LeMay, Pommerening, and Marshall (2009), who found that there were higher germination and survival rates of interior Douglas‐fir near big trees on moisture‐limited sites. This is further evidence that site‐specific silviculture is needed. Our study considers 3‐year‐old (two growing seasons in the field) seedlings, and the results should only be applied to early establishment.

Our findings also support the use of innovative silvicultural practices and provenance selection that are tailored to a specific ecosystem. There was a large amount of variation attributed to the location of our study sites (Tables S4–S8). This highlights the importance of spatially situating management plans to consider site‐specific conditions of the forest, not just the larger climate of the area.

Spatially situating management plans means understanding the ecosystem processes of a certain stand created by natural disturbance patterns. Interior Douglas‐fir ecosystems predominantly experience natural disturbances that result in low‐ to moderate‐sized openings in the canopy (Hessburg et al. 2019; Leclerc, Daniels, and Carroll 2021). Silviculture systems that mimic natural disturbance, such as shelterwood, variable retention, group retention, and patch cutting, have all been considered as ways to help enhance forest regeneration (Mitchell and Beese 2002; Day, Koot, and Wiensczyk 2011; Beese et al. 2019; Pommerening 2023). Leaving residual trees in these areas post‐harvest as climate changes will provide shelter for planted seedlings to survive increasingly extreme weather events (Franklin et al. 2002). As the climate changes, using systems that have inherent benefits to regeneration may be prudent.

## 5 Conclusions

Our study found that increasing overstory retention was beneficial to seedling establishment without impacting height growth when seedlings were moved to climates with greater aridity or fewer frost‐free days. By contrast, seedlings transferred to more favorable (warmer and wetter) climates performed better with lower levels of overstory retention. To our knowledge, this is the first study that has examined how assisted population migration of seedlings can be augmented by careful design of silviculture systems. Regeneration success can be substantially improved where it is combined with site‐specific degrees of overstory retention. Our results demonstrate that regenerating forests in a changing climate must be done with an understanding of stand‐level microclimates within a broader ecosystem. Continuing to manage forests under the assumption of a stable climate creates risk for the future health and productivity of forests. We suggest that maintaining overstory trees will protect the seedlings and assist in early establishment and regeneration of stands. As climate continues to change and novel climatic envelopes emerge, it will be increasingly important to know how best to protect regenerating seedlings against climatic maladaptation.

## Author Contributions

**Thomson C. Harris:** data curation, formal analysis, investigation, methodology, visualization, writing – original draft, writing – review and editing. **Suzanne W. Simard:** conceptualization, data curation, funding acquisition, investigation, methodology, project administration, resources, supervision, validation, writing – review and editing. **W. Jean Roach:** conceptualization, data curation, funding acquisition, methodology, project administration, resources, supervision, writing – review and editing. **Erin M. Miller:** writing – original draft, writing – review and editing.

## Conflicts of Interest

The authors declare no conflicts of interest.

## Supporting information


Data S1.

## Data Availability

The data that support the findings of this study are openly available in Zenodo at https://doi.org/10.5281/zenodo.13363440. Climate data was obtained from the ClimateWNA v7.40 model at https://climatena.ca/.
