# Supplementary material for: The Interactive Role of Climatic Transfer Distance and Overstory Retention on Douglas‐Fir Seedling Survival and Height Growth in Interior British Columbia
Source: Glob Chang Biol. 2025 Jan 24;31(1):e70027. doi: 10.1111/gcb.70027 (PMC11758759; doi:10.1111/gcb.70027)
Supplement: Supplementary file 1 — Data S1. [file GCB-31-e70027-s001.docx]

**Treatment Block Layout**

**
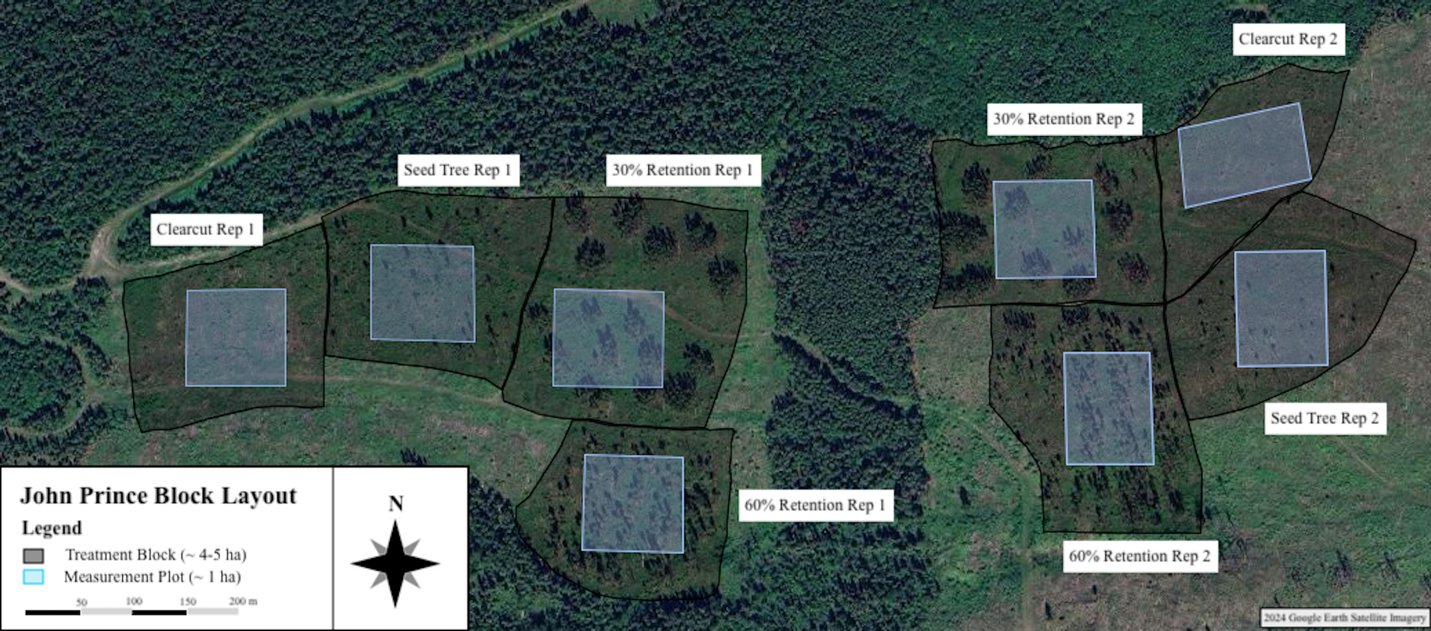
**

**Figure 1.** Ariel view of the treatment block layout at the John Prince location. Centrally located measurement plots are displayed in blue. Centrality of the measurement plots are constrained by the configuration of the treatment block.

**Model Evaluation**

Presented here are eight tables showing the testing procedure for the four families of models tested and the selected model output: Survival – Harvest Method (Table 1, 5), Survival – Crown Closure (Table 2, 6), Height – Harvest Method (Table 3, 7), and Height – Crown Closure (Table 4, 8). For both survival models mean annual relative humidity transfer distance (RH_td_, %) is the climatic variable presented. Height models present mean annual precipitation as snow transfer distance (PAS_td_, mm). These are the best performing (highest scaled parameter estimates, see Fig. 5 main text) climatic transfer distance (CTD) variables for the respective models. As there are nine climatic variables tested for each family of models (for a total of 116 models) we only present one for each family here. The log-likelihood, AIC, marginal R^2^, and conditional R^2^ are similar across each family of model. The reason for including these tables is to give insight and transparency to the model selection and evaluation process. Below is an in-depth explanation of the model selection process for the Survival – Harvest Method family using RH_td_ as an example CTD variable.

Models were primarily evaluated based on the p-value of the likelihood ratio test with an alpha value of α = 0.05. Using the Table 1 as an example, the testing hierarchy was as follows. The Harvest Method and RH_td_ models (model number 2 and 3) were tested against the null model (model number 1), obtaining p-values of p = 0.27 and p < 0.001 respectively. The RH_td_ – Harvest Method model (model number 4) was tested against the RH_td_ model (model number 3) obtaining a p-value of p = 0.31. The RH_td_ – Harvest Method interaction model (model number 5) was tested against both the RH_td_ – Harvest Method model and the RH_td_ model (model number 4 and 3) as Harvest Method was not a significant inclusion. These tests resulted in a p-values of 0.004 and 0.01 respectively. The result of these tests lead to selecting the The RH_td_ – Harvest Method interaction model (model number 5) as the best model for this climatic transfer distance variable. The inclusion of the test of model number 5 against model number 3 is only done for the Survival models that include Harvest Method as it is not relevant for other models.

**Table 1.** Model statements, goodness-of-fit, and likelihood ratio test results for the Survival – Harvest Method family of models using RH_td_ as an example climatic transfer distance variable.

| **Model Number** | **Survival Model Statement^ab^** | **Log-Likelihood** | **AIC** | **Marginal/Cond. R^2^** | **df** | **χ^2^** | **p-value** |
| --- | --- | --- | --- | --- | --- | --- | --- |
| 1 | ~ 1 | - 3799.1 | 7608.2 | 0.000 / 0.450 | 5 | – | |
| 2 | ~ Harvest Method | -3797.2 | 7610.3 | 0.009 / 0.449 | 8 | 3.9 | 0.27 |
| 3 | ~ RH_td_^cd^ | -3769.8 | 7551.7 | 0.111 / 0.416 | 6 | **58.5** | **<0.001** |
| 4 | ~ RH_td_ + Harvest Method | -3768.1 | 7554.1 | 0.120 / 0.417 | 9 | 3.6 | 0.31 |
| **5^e^** | **~ RH_td_ + Harvest Method + RH_td_ x Harvest Method** | **-3761.4** | **7546.8** | **0.137 / 0.413** | **12** | **13.3 / 16.9** | **0.004 / 0.01** |

^a^ Model Statement here exclude random effects for brevity. Full model statement example:

*Survival ~ scaled(RH_td_) + Harvest Method + scaled(RH_td_) x Harvest Method + (Location/Block/Plot/Splitplot)*

^b^ Final model selection is bolded

^c^ Test shown against the null model (model #1) not the previous model (model #2).

^d^ RH_td_ = scaled mean annual relative humidity transfer distance.

^e^  Tested against both model 4 and model 3 as Harvest Method was not individually significant.

**Table 2.** Model statements, goodness-of-fit, and likelihood ratio test results for the Survival – Crown Closure family of models using RH_td_ as an example climatic transfer distance variable.

| **Model Number** | **Survival Model Statement^ab^** | **Log-Likelihood** | **AIC** | **Marginal/Cond. R^2^** | **df** | **χ^2^** | **p-value** |
| --- | --- | --- | --- | --- | --- | --- | --- |
| 1 | ~ 1 | -3799.1 | 7608.2 | 0.000 / 0.450 | 5 | – | |
| 2 | ~ Canopy Cover^e^ | -3749.4 | 7510.7 | 0.022 / 0.459 | 6 | **99.5** | **<0.001** |
| 3 | ~ RH_td_^cd^ | -3769.8 | 7551.7 | 0.111 / 0.416 | 6 | **58.5** | **<0.001** |
| 4 | ~ RH_td_ + Canopy Cover | -3720.3 | 7454.6 | 0.134 / 0.428 | 7 | **99.1** | **<0.001** |
| **5** | **~ RH_td_ + Canopy Cover + RH_td_ x Canopy Cover** | **-3714.2** | **7444.5** | **0.141 / 0.425** | **8** | **12.1** | **<0.001** |

^a^ Model Statement here exclude random effects for brevity. Full model statement example:

*Survival ~ scaled(RH_td_) + sqrt(Canopy Cover) + scaled(RH_td_) x sqrt(Canopy Cover) + (Location/Block/Plot/Splitplot)*

^b^ Final model selection is bolded

^c^ Tested against the null model (model #1) not the previous model (model #2).

^d^ RH_td_ = scaled mean annual relative humidity transfer distance.

^e^ Canopy cover term is square rooted

**Table 3.** Model statements, goodness-of-fit, and likelihood ratio test results for the Height – Harvest Method family of models using PAS_td_ as an example climatic transfer distance variable.

| **Model Number** | **Height Model Statement^ab^** | **Log-Likelihood** | **AIC** | **Marginal/Cond. R^2^** | **df** | **χ^2^** | **p-value** |
| --- | --- | --- | --- | --- | --- | --- | --- |
| 1 | ~ 1 | -2577.2 | 5166.3 | 0.000 / 0.369 | 6 | – | |
| 2 | ~ Harvest Method^c^ | -2566.8 | 5151.6 | 0.048 / 0.369 | 9 | **20.7** | **<0.001** |
| 3 | ~ PAS_td_^cd^ | -2562.7 | 5139.3 | 0.053 / 0.390 | 7 | **29.0** | **<0.001** |
| **4** | **~ PAS_td_ + Harvest Method** | **-2552.6** | **5125.2** | **0.095 / 0.388** | **10** | **20.1** | **<0.001** |
| 5 | ~ PAS_td_ + Harvest Method + PAS_td_ x Harvest Method | -2551.5 | 5129.0 | 0.097 / 0.388 | 13 | 2.2 | 0.53 |

^a^ Model Statement here exclude random effects for brevity. Full model statement example:

*ln(Height) ~ scaled(PAS_td_) + Harvest Method + scaled(PAS_td_) x Harvest Method + (Location/Block/Plot/Splitplot)*

^b^ Final model selection is bolded

^c^ Tested against the null model (model #1) not the previous model (model #2).

^d^ PAS_td_ = scaled mean annual precipitation as snow transfer distance.

**Table 4.** Model statements, goodness-of-fit, and likelihood ratio test results for the Height – Crown Closure family of models using PAS_td_ as an example climatic transfer distance variable.

| **Model Number** | **Height Model Statement^ab^** | **Log-Likelihood** | **AIC** | **Marginal/Cond. R^2^** | **df** | **χ^2^** | **p-value** |
| --- | --- | --- | --- | --- | --- | --- | --- |
| 1 | ~ 1 | -2577.2 | 5166.3 | 0.000 / 0.369 | 6 | – | |
| 2 | ~ Canopy Cover | -2439.6 | 4893.2 | 0.047 / 0.381 | 7 | 275.1 | **<0.001** |
| 3 | ~ PAS_td_^cd^ | -2562.7 | 5139.3 | 0.053 / 0.390 | 7 | 29.0 | **<0.001** |
| **4** | **~ PAS_td_ + Canopy Cover** | **-2425.5** | **4867.1** | 0.102 / 0.407 | **8** | **274.2** | **<0.001** |
| 5 | ~ PAS_td_^c^ + Canopy Cover + PAS_td_^c^ x Canopy Cover | -2425.5 | 4869.0 | 0.102 / 0.407 | 9 | 0.03 | 0.87 |

^a^ Model Statement here exclude random effects for brevity. Full model statement example:

*ln(Height) ~ scaled(PAS_td_) + sqrt(Canopy Cover) + scaled(PAS_td_) x sqrt(Canopy Cover) + (Location/Block/Plot/Splitplot)*

^b^ Final model selection is bolded

^c^ Tested against the null model (model #1) not the previous model (model #2).

^d^ PAS_td_ = scaled mean annual precipitation as snow transfer distance.

**Table 5.** Model output from the survival interaction model of relative humidity transfer distance (RH_td_, %) and harvest method.

**Table 6.** Model output from the survival interaction model of scaled relative humidity transfer (RH_td_, %) distance and square root crown closure.

**Table 7.** Model output from the height interaction model of precipitation as snow transfer distance (PAS_td_, mm) and harvest method.

**Table 8.** Model output from the height interaction model of precipitation as snow transfer distance (PAS_td_, mm) and canopy closure.

**Model Graphs**


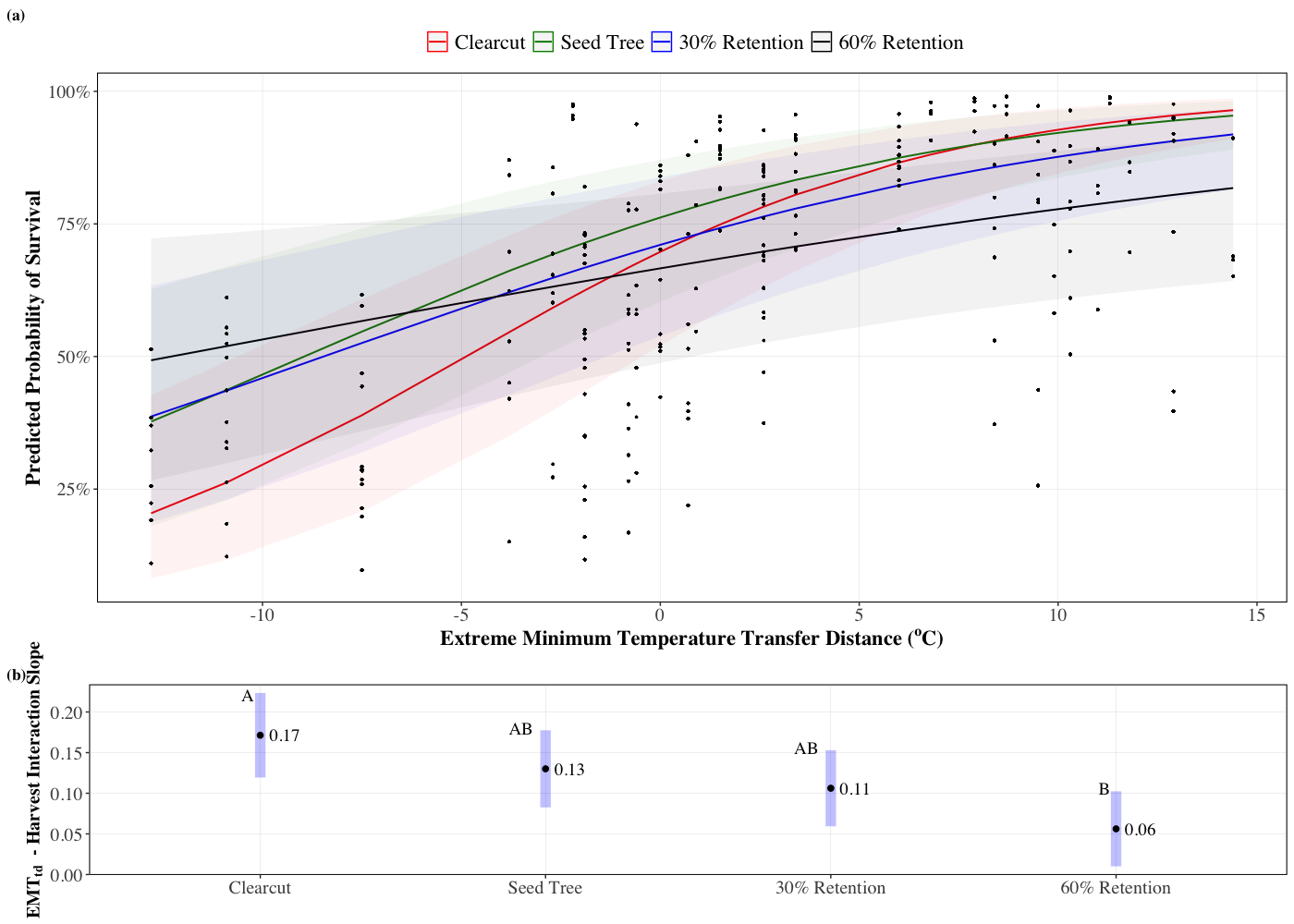


**Figure 2.** a): Estimated probability of survival^a^ (%) against the extreme minimum temperature over 30 years transfer distance (EMT_td_, ^o^C) (Marginal and Conditional R^2^ of 0.111 and 0.406 respectively) for each of the harvest methods. b): Estimated Marginal slopes EMT_td_ for each harvest methods. Different letters indicate significant differences. The clearcut and 60% retention treatment interaction slopes are significantly different for the EMT_td_ model with p = 0.005.

^a^ *Survival ~ Transfer Distance + Harvest + Transfer Distance*Harvest + (Location/Block/Plot/Splitplot)*


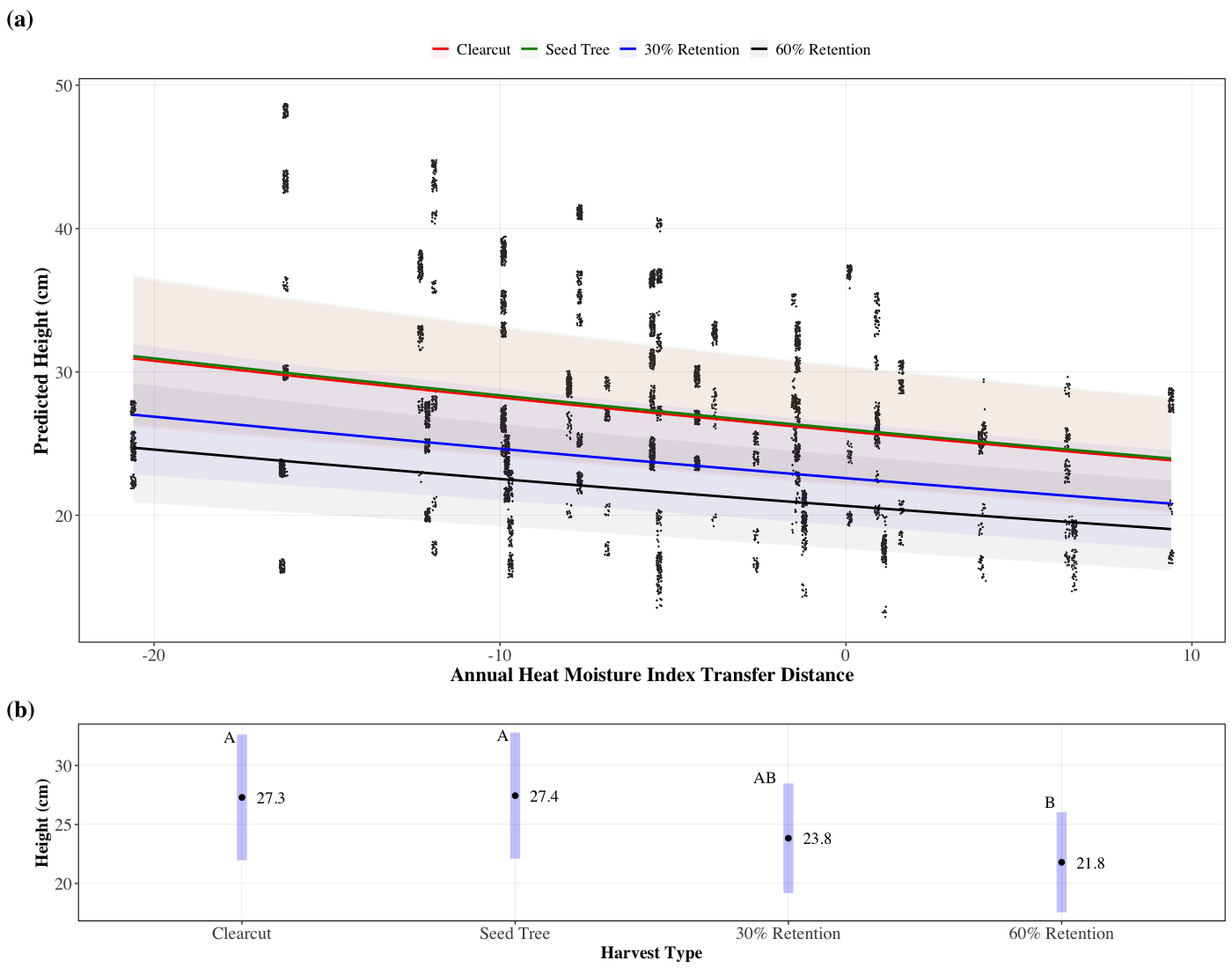


**Figure 3.** a): Predicted height^a^ (cm) against annual heat moisture index transfer distance (AHM_td_) for each harvest method. b): Estimated Marginal means for AHM_td_ for each harvest method. Different letters indicate significant differences. (Pairwise test, clearcut - 60% retention p-value = 0.015, Seed tree - 60% retention p-value = 0.011). Marginal and Conditional R^2^ of 0.064 and 0.359, respectively.

^a^ *ln(height) ~ Transfer Distance + Harvest + Transfer Distance*Harvest + (Location/Block/Plot/Splitplot)*


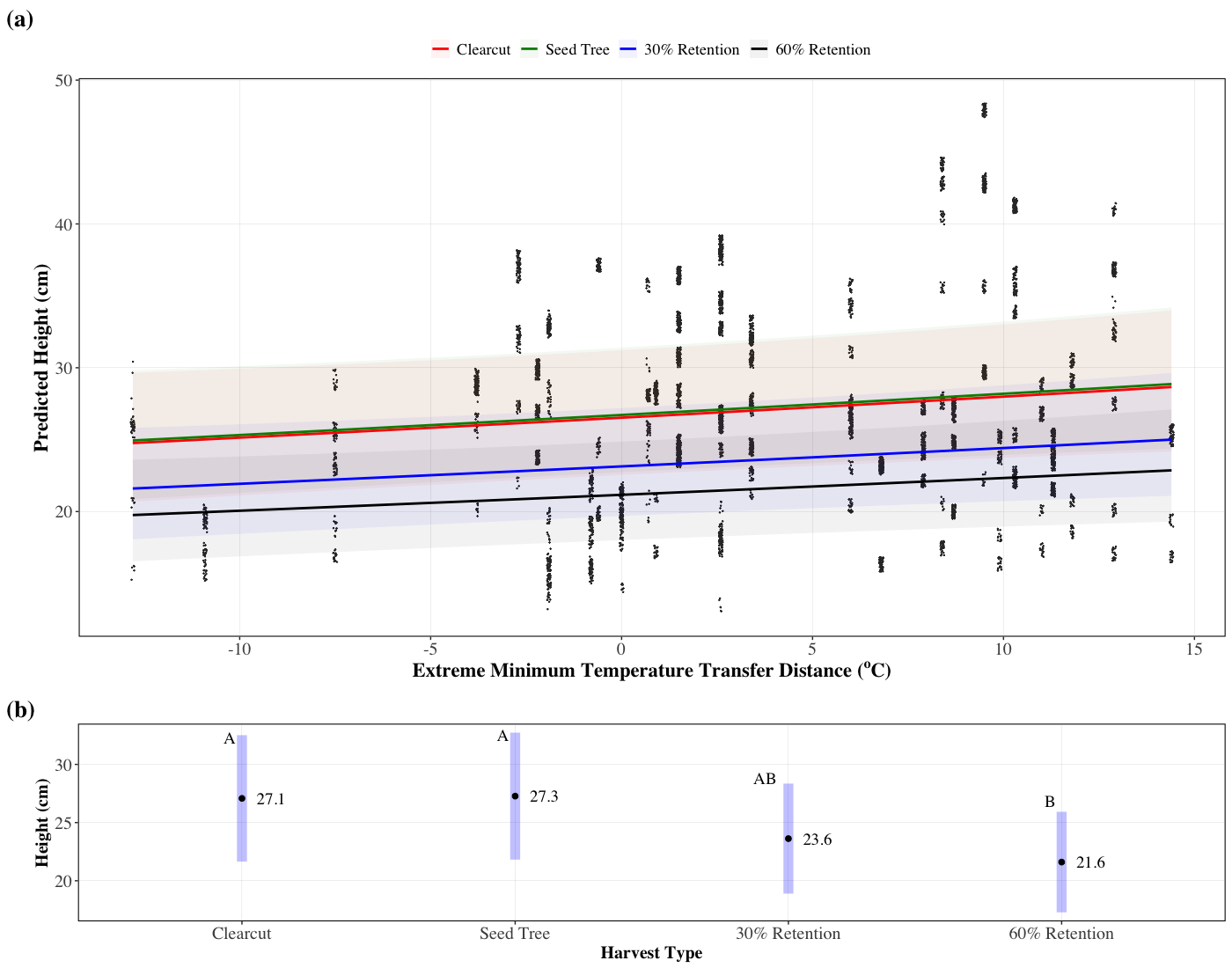


**Figure 4.** a): Predicted height^a^ (cm) extreme minimum temperature over 30 years transfer distance (EMT_td_, ^o^C) for each harvest method. b): Estimated Marginal means for EMT_td_ for each harvest method. Different letters indicate significant differences. (Pairwise test, clearcut - 60% retention p-value = 0.015, Seed tree - 60% retention p-value = 0.011). Marginal and Conditional R^2^ of 0.055 and 0.365, respectively.

^a^ *ln(height) ~ Transfer Distance + Harvest + Transfer Distance*Harvest + (Location/Block/Plot/Splitplot)*


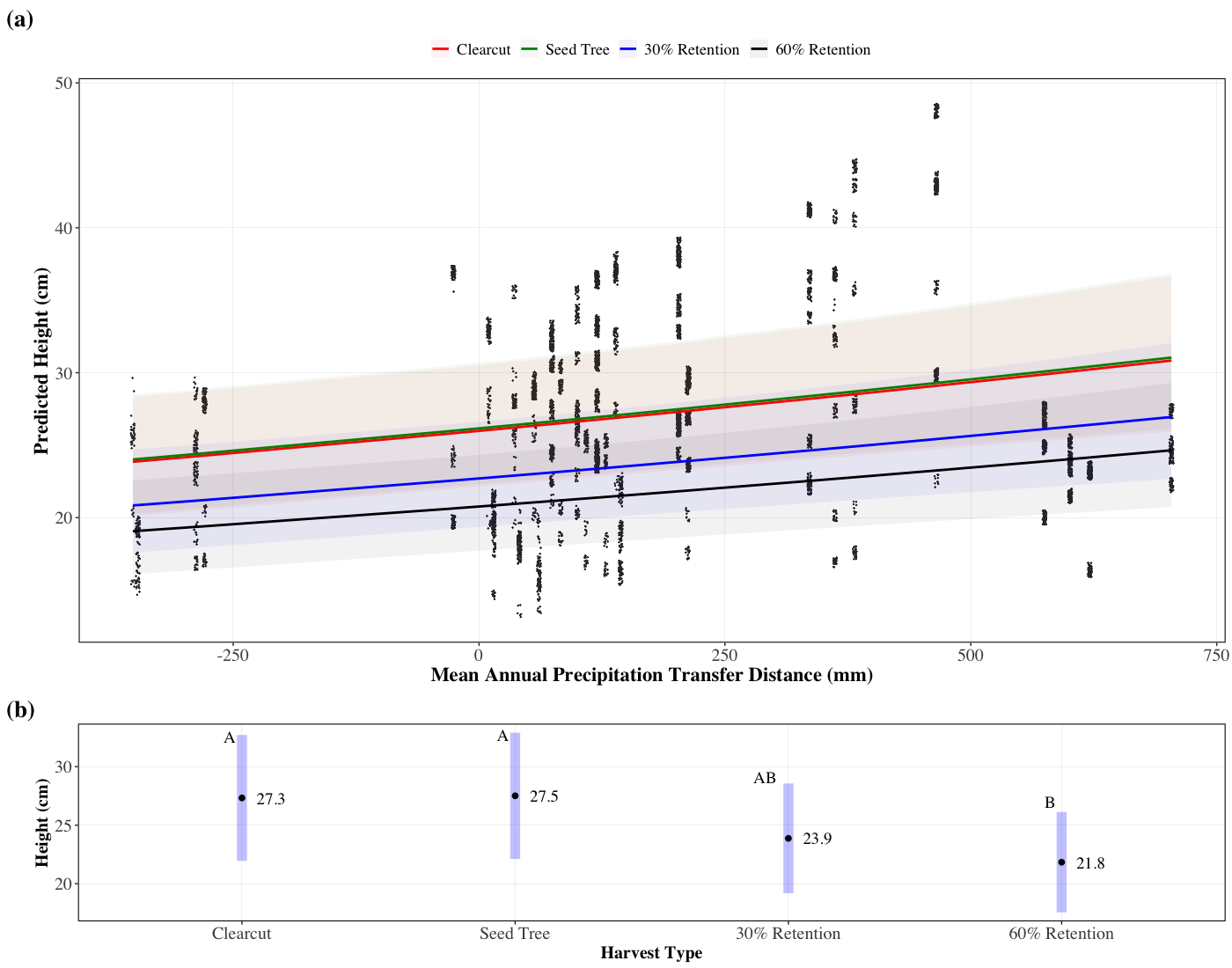


**Figure 5.** a): Predicted height^a^ (cm) against mean annual precipitation transfer distance (MAP_td_, mm) for each harvest method. b): Estimated Marginal means for MAP_td_ for each harvest method. Different letters indicate significant differences. (Pairwise test, clearcut - 60% retention p-value = 0.015, Seed tree - 60% retention p-value = 0.011). Marginal and Conditional R^2^ of 0.066 and 0.365, respectively.

^a^ *ln(height) ~ Transfer Distance + Harvest + Transfer Distance*Harvest + (Location/Block/Plot/Splitplot)*


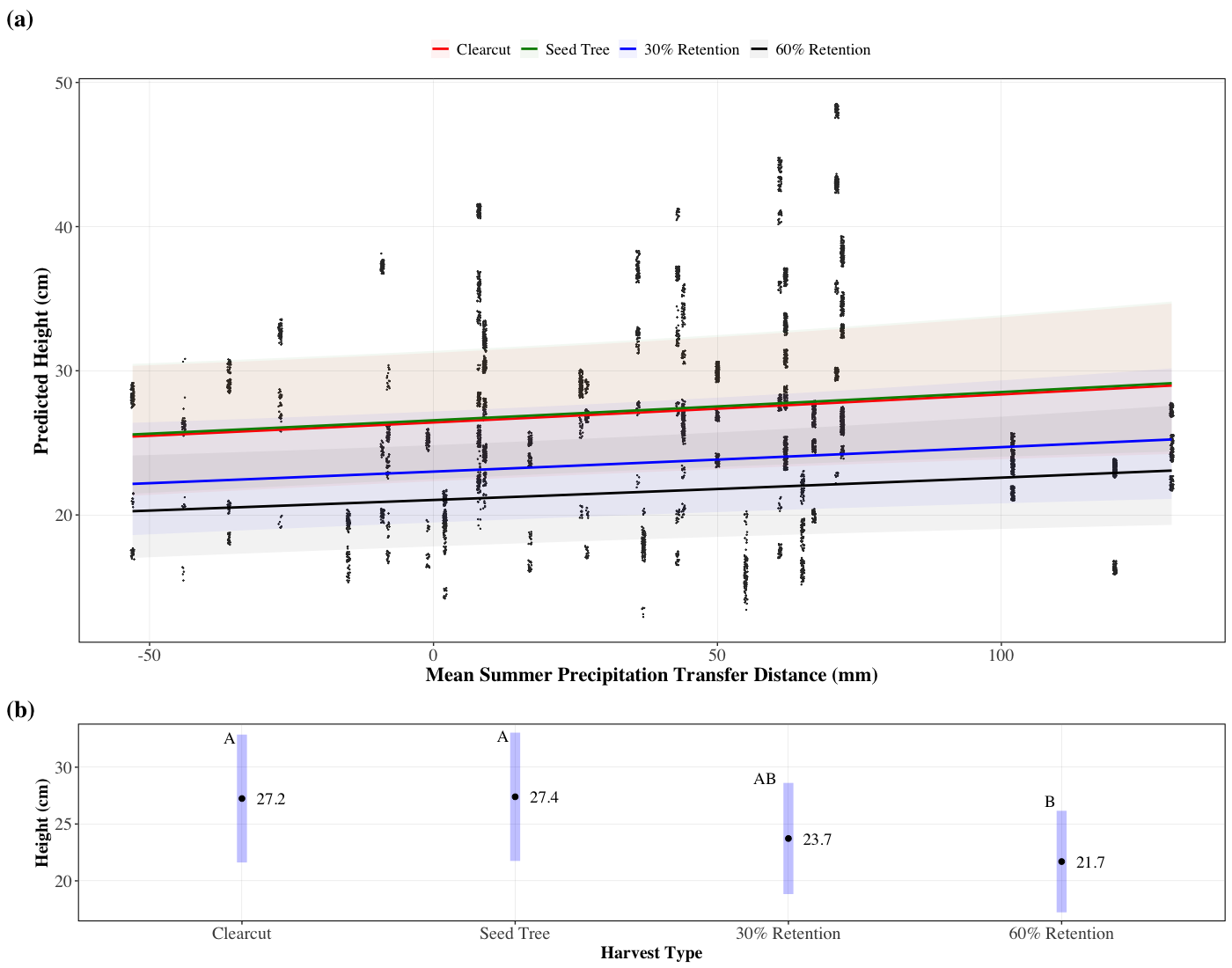


**Figure 6.** a): Predicted height^a^ (cm) against mean summer precipitation transfer distance (MSP_td_, mm) for each treatment type. b): Estimated Marginal means for MSP_td_ for each harvest method. Different letters indicate significant differences. (Pairwise test, clearcut - 60% retention p-value = 0.015, Seed tree - 60% retention p-value = 0.011). Marginal and Conditional R^2^ of 0.051 and 0.359, respectively.

^a^ *ln(height) ~ Transfer Distance + Harvest + Transfer Distance*Harvest + (Location/Block/Plot/Splitplot)*


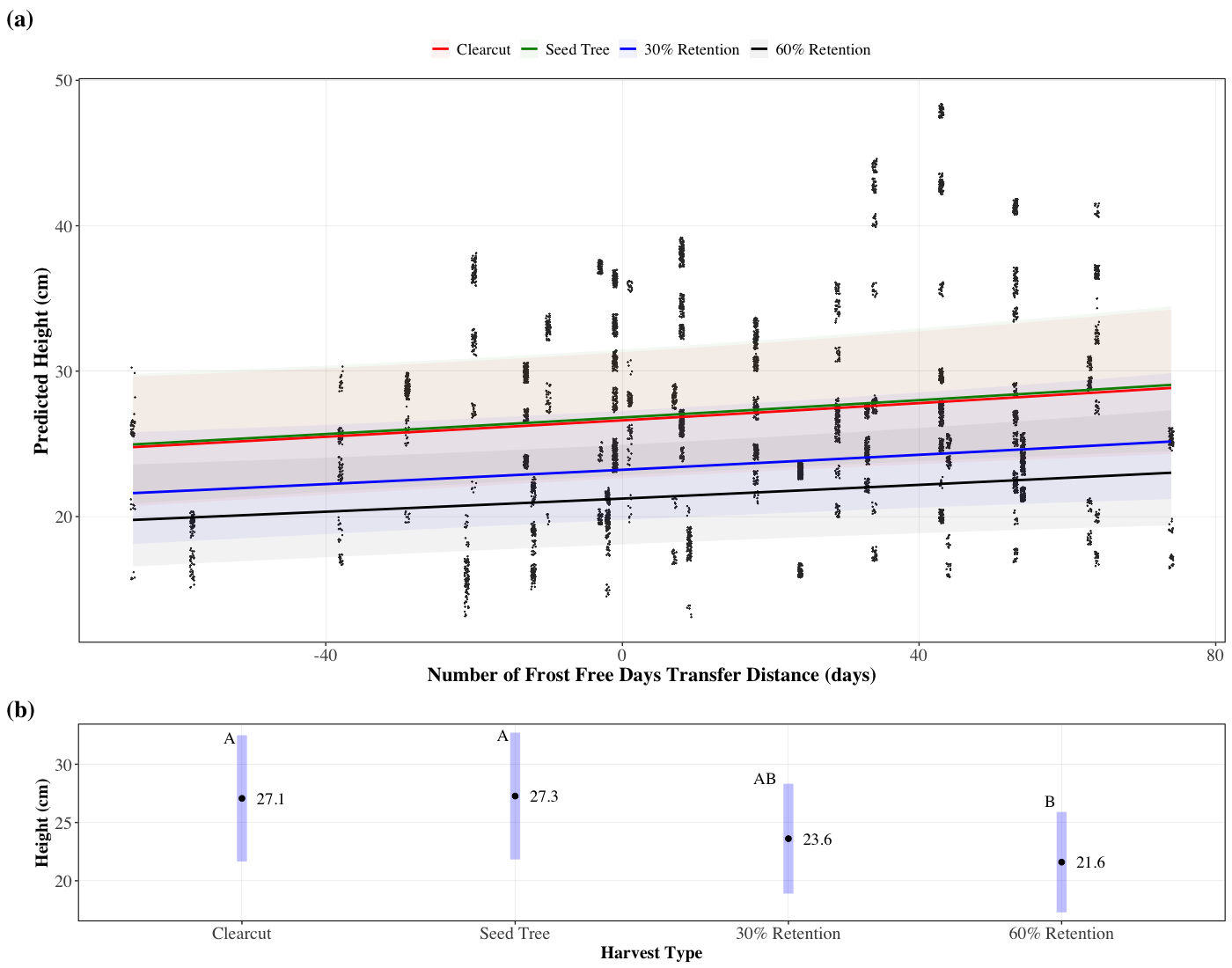


**Figure 7.** a): Predicted height^a^ (cm) against number of frost-free days transfer distance (NFFD_td_, days) for each harvest method. b): Estimated Marginal means for NFFD_td_ for each harvest method. Different letters indicate significant differences. (Pairwise test, clearcut - 60% retention p-value = 0.015, Seed tree - 60% retention p-value = 0.011). Marginal and Conditional R^2^ of 0.055 and 0.365, respectively.

^a^ *ln(height) ~ Transfer Distance + Harvest + Transfer Distance*Harvest + (Location/Block/Plot/Splitplot)*


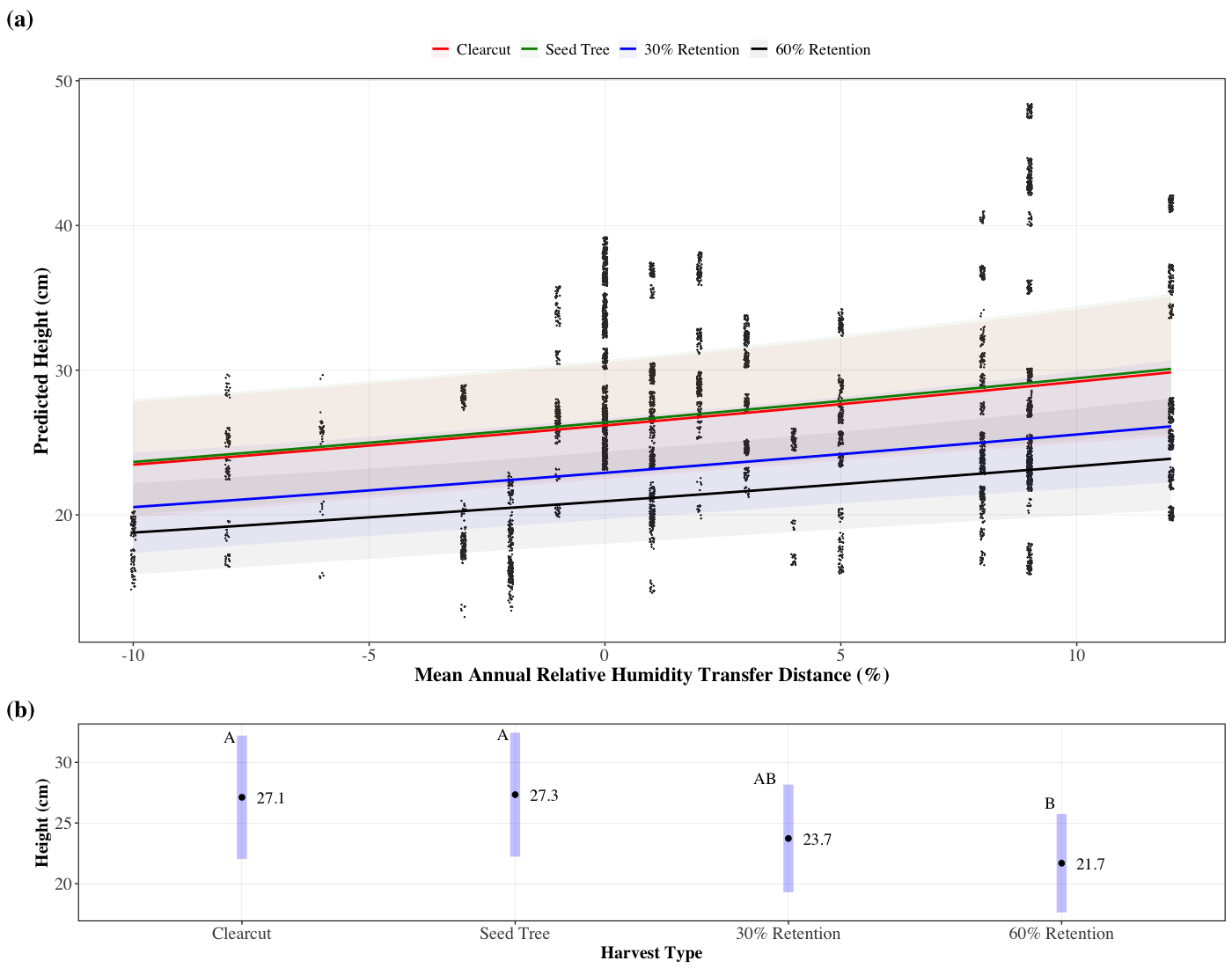


**Figure 8.** a): Predicted height^a^ (cm) against mean annual relative humidity transfer distance (RH_td_, %) for each harvest method. b): Estimated Marginal means for RH_td_ for each harvest method. Different letters indicate significant differences. (Pairwise test, clearcut - 60% retention p-value = 0.015, Seed tree - 60% retention p-value = 0.011). Marginal and Conditional R^2^ of 0.066 and 0.355, respectively.

^a^ *ln(height) ~ Transfer Distance + Harvest + Transfer Distance*Harvest + (Location/Block/Plot/Splitplot)*


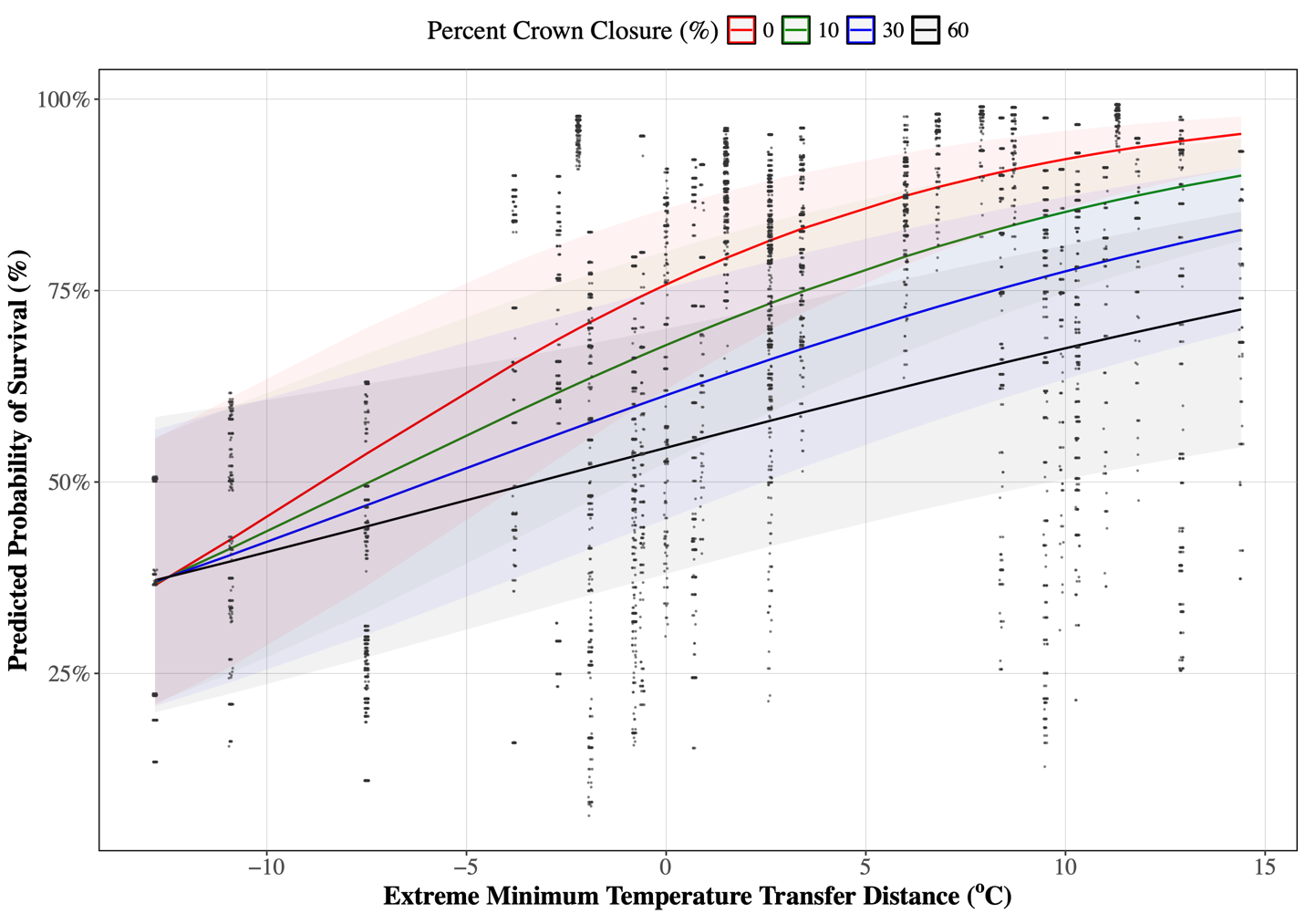


**Figure 9.** Predicted probability of survival^a^ (%) against extreme minimum temperature over 30 years transfer distance (EMT_td_, ^o^C) for different levels of crown closure (%). Marginal and Conditional R^2^ of 0.123 and 0.413 respectively.

^a^ *Survival ~ Transfer Distance + Crown Closure + Transfer Distance*Crown Closure + (Location/Block/Plot/Splitplot)*


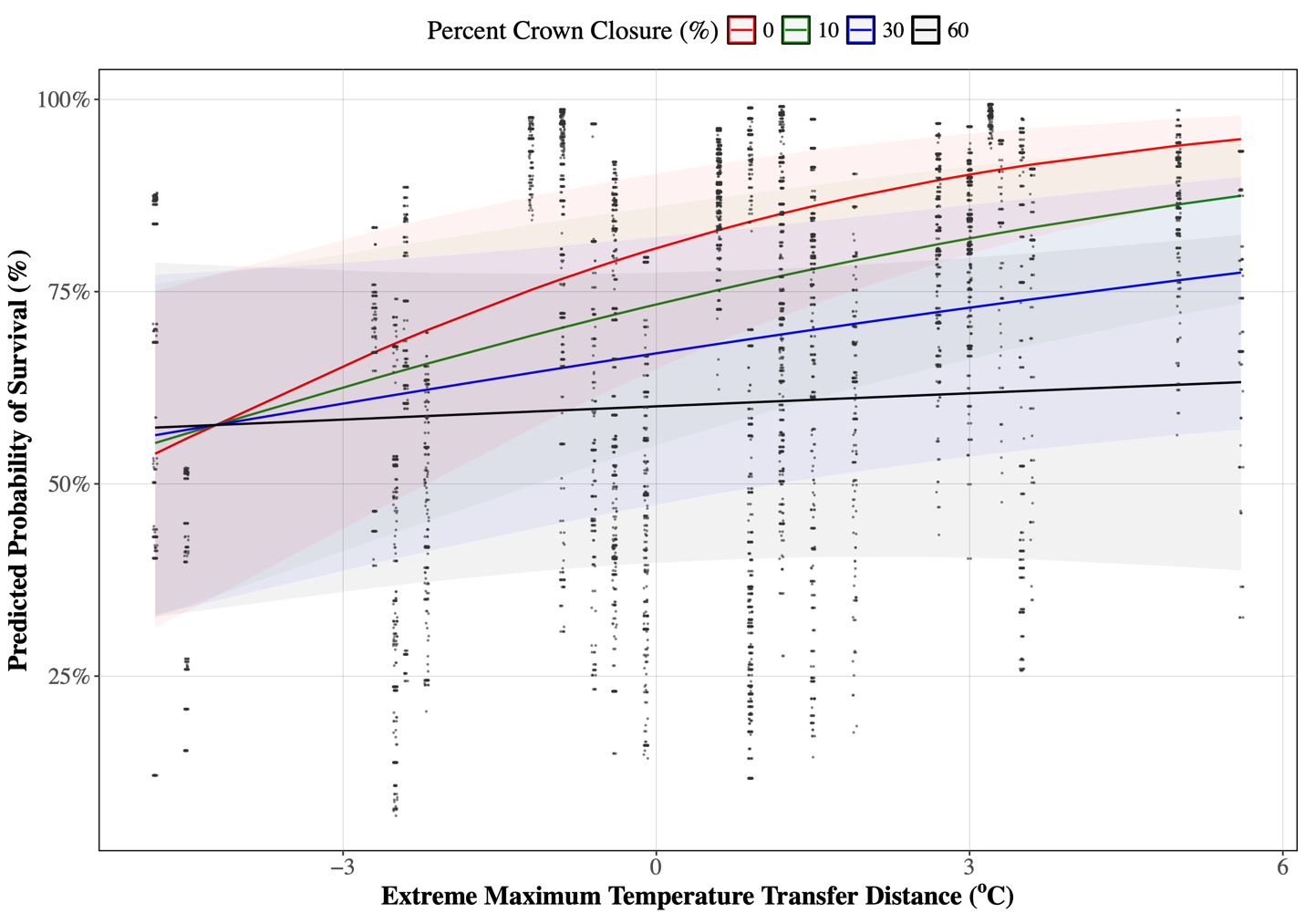


**Figure 10.** Predicted probability of survival^a^ (%) against extreme maximum temperature over 30 years transfer distance (EXT_td_, ^o^C) for different levels of crown closure (%). Marginal and Conditional R^2^ of 0.070 and 0.444 respectively.

^a^ *Survival ~ Transfer Distance + Crown Closure + Transfer Distance*Crown Closure + (Location/Block/Plot/Splitplot)*


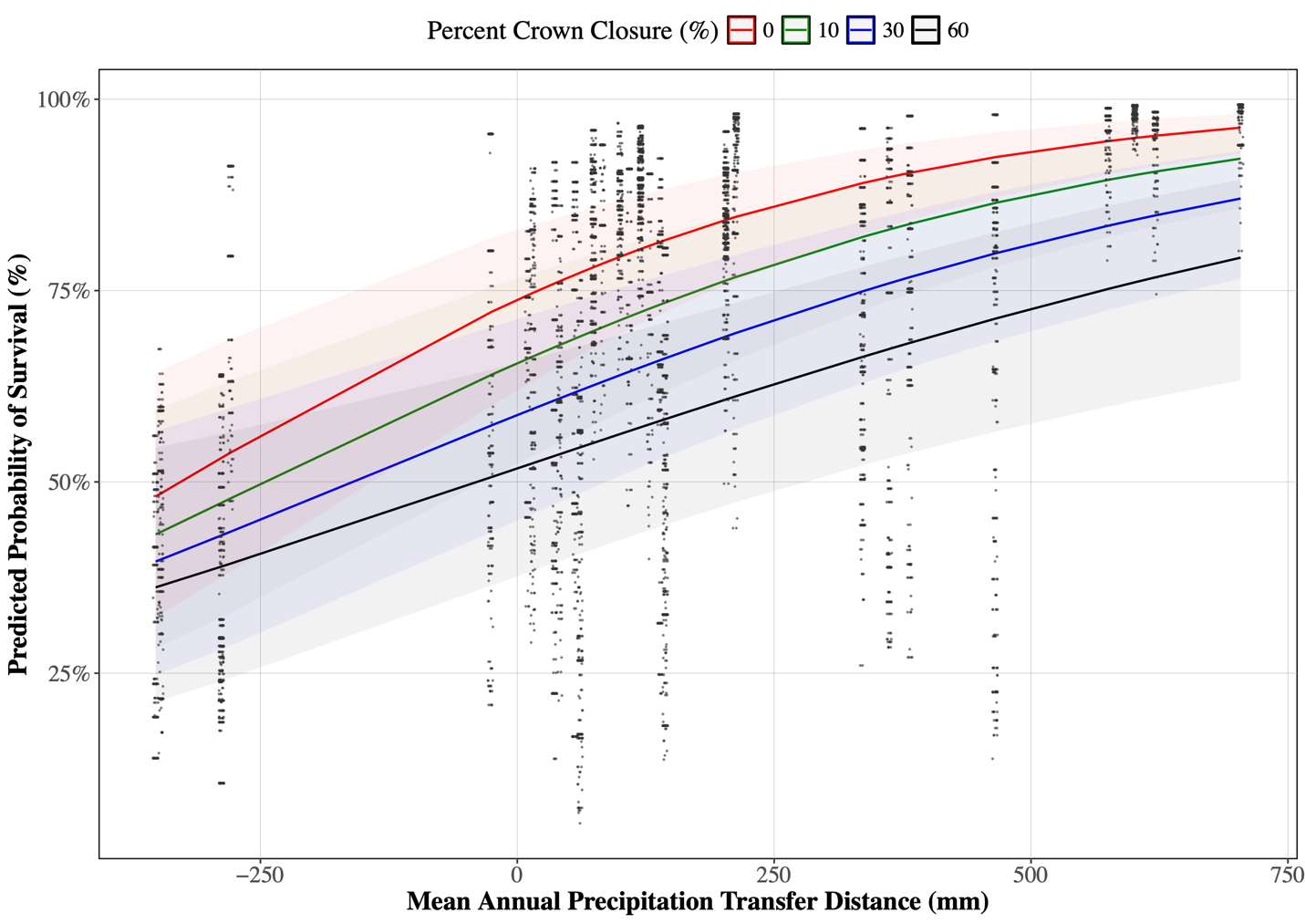


**Figure 11.** Predicted probability of survival^a^ (%) against mean annual precipitation transfer distance (MAP_td_, mm) for different levels of crown closure (%). Marginal and Conditional R^2^ of 0.128 and 0.400 respectively.

^a^ *Survival ~ Transfer Distance + Crown Closure + Transfer Distance*Crown Closure + (Location/Block/Plot/Splitplot)*


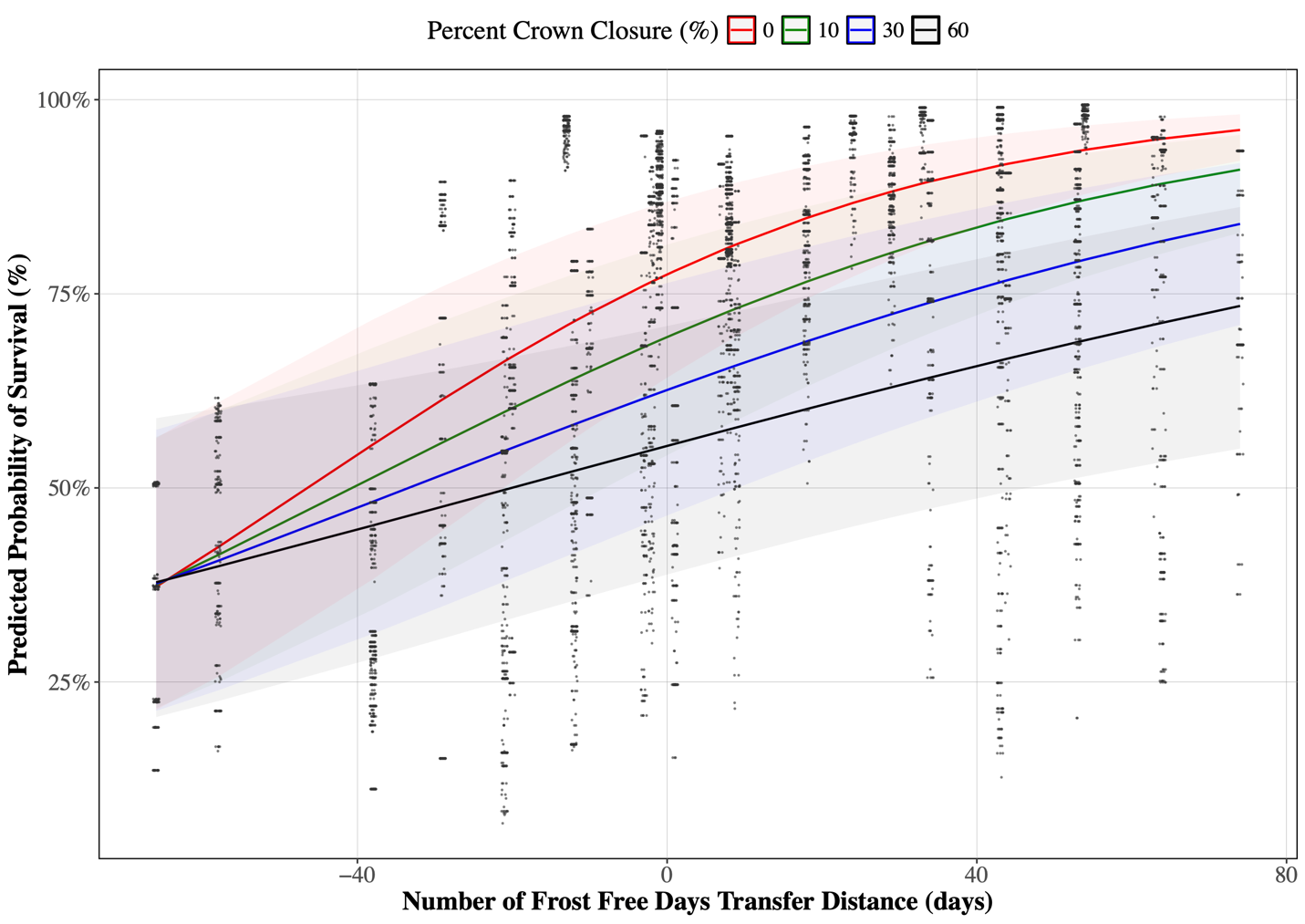


**Figure 12.** Predicted probability of survival^a^ (%) against number of frost-free days transfer distance (NFFD_td_, days) for different levels of crown closure (%). Marginal and Conditional R^2^ of 0.128 and 0.419 respectively.

^a^ *Survival ~ Transfer Distance + Crown Closure + Transfer Distance*Crown Closure + (Location/Block/Plot/Splitplot)*


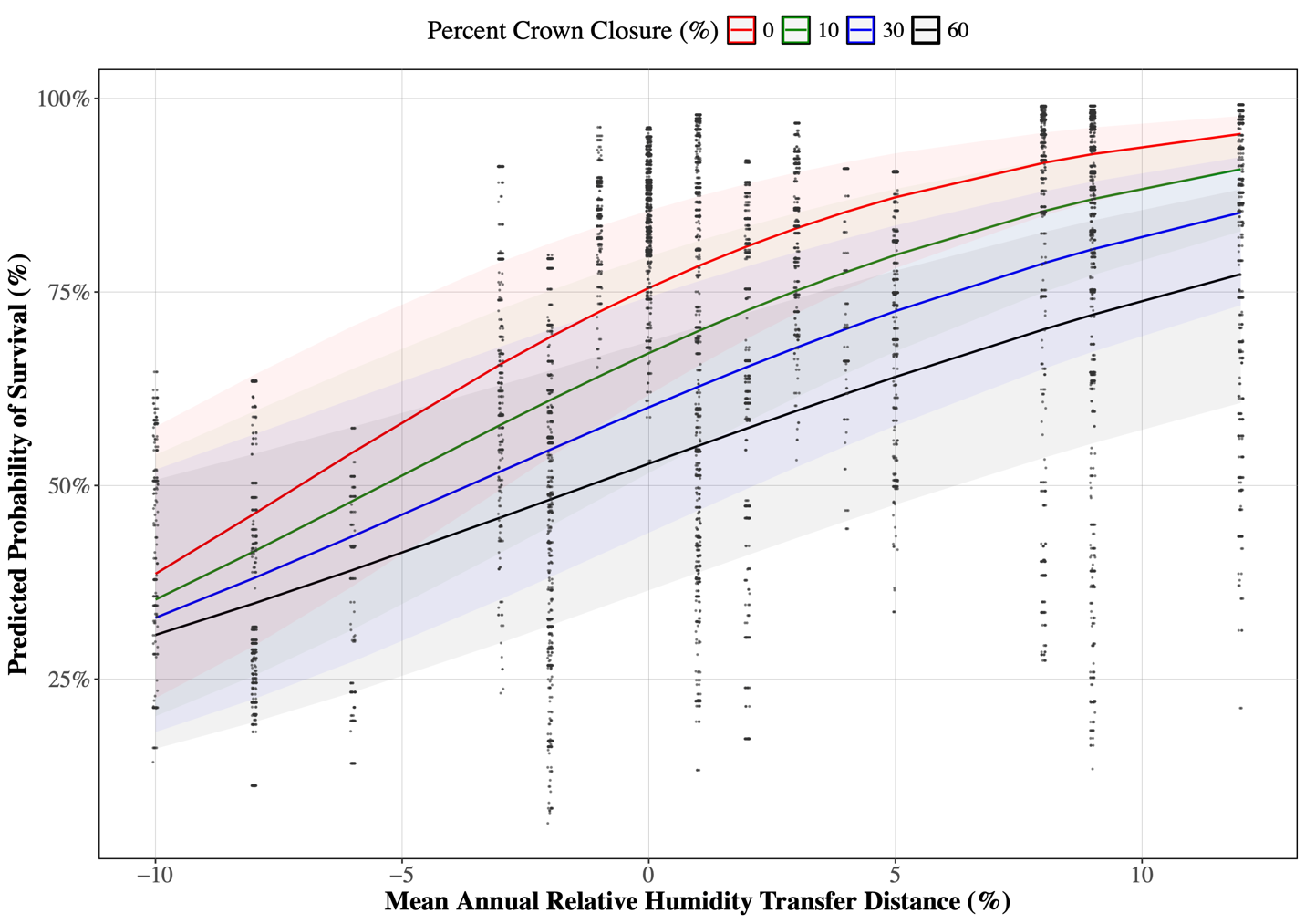


**Figure 13.** Predicted probability of survival^a^ (%) against mean annual relative humidity transfer distance (RH_td_, %) for different levels of crown closure (%). Marginal and Conditional R^2^ of 0.141 and 0.425 respectively.

^a^ *Survival ~ Transfer Distance + Crown Closure + Transfer Distance*Crown Closure + (Location/Block/Plot/Splitplot)*


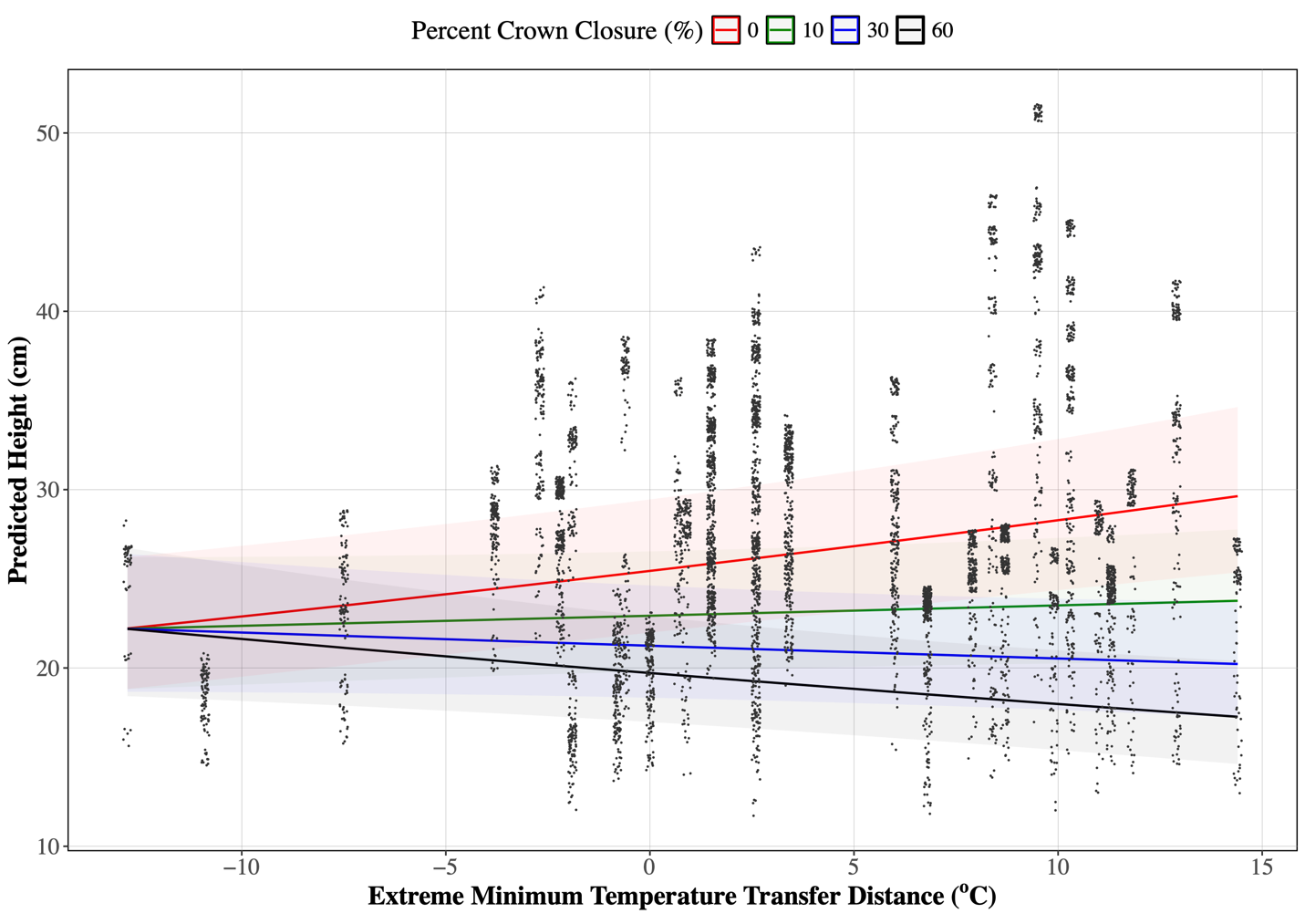


**Figure 14.** Predicted height^a^ (cm) against extreme minimum temperature over 30 years transfer distance (EMT_td_, ^o^C) for different levels of crown closure. Marginal and Conditional R^2^ of 0.068 and 0.374 respectively.

^a^ *ln(height ) ~ Transfer Distance + Crown Closure + Transfer Distance*Crown Closure + (Location/Block/Plot/Splitplot)*

**
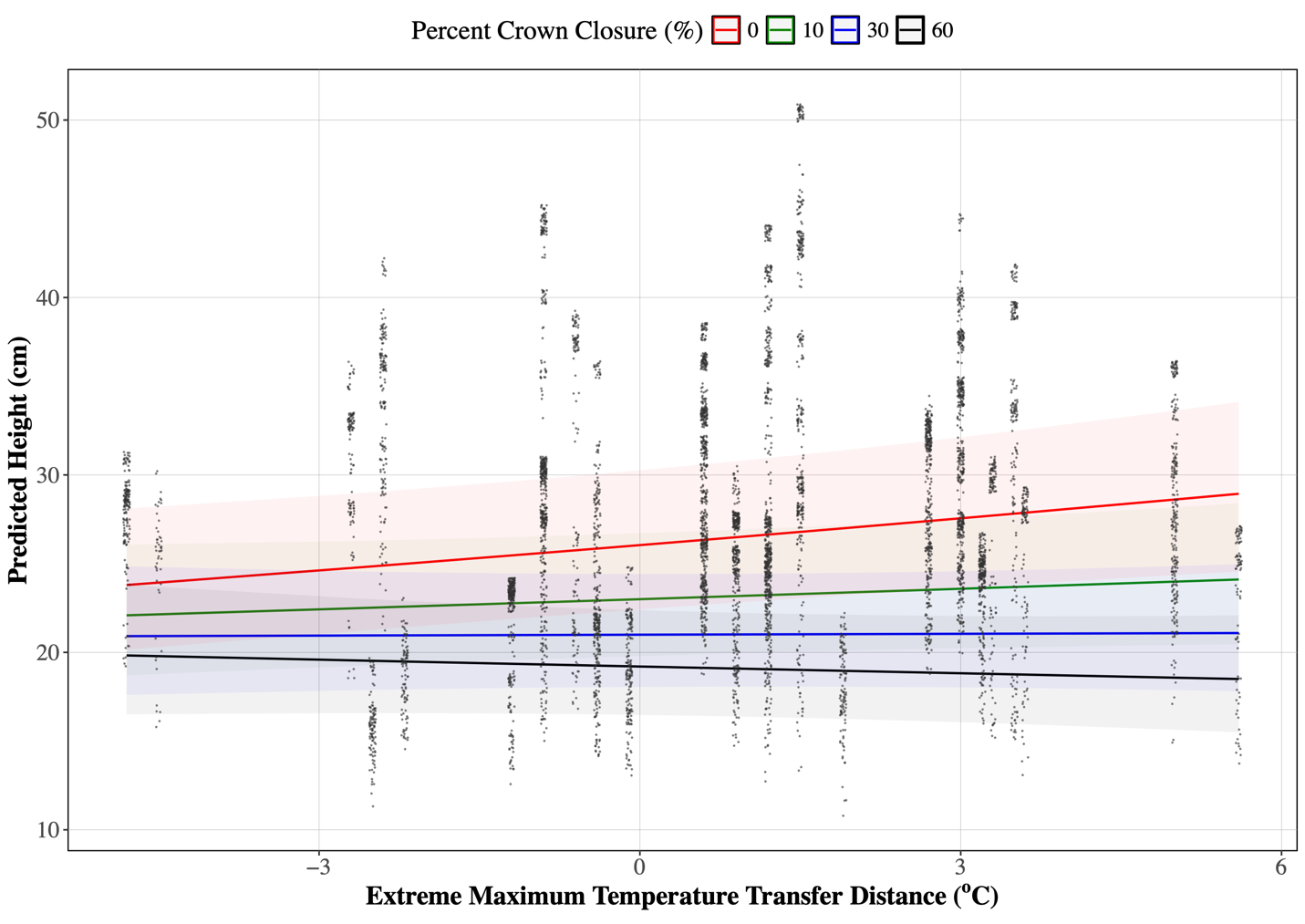
**

**Figure 15.** Predicted height^a^ (cm) against extreme maximum temperature over 30 years transfer distance (EXT_td_, ^o^C) for different levels of crown closure (%). Marginal and Conditional R^2^ of 0.065 and 0.376 respectively.

^a^ *ln(height ) ~ Transfer Distance + Crown Closure + Transfer Distance*Crown Closure + (Location/Block/Plot/Splitplot)*

**
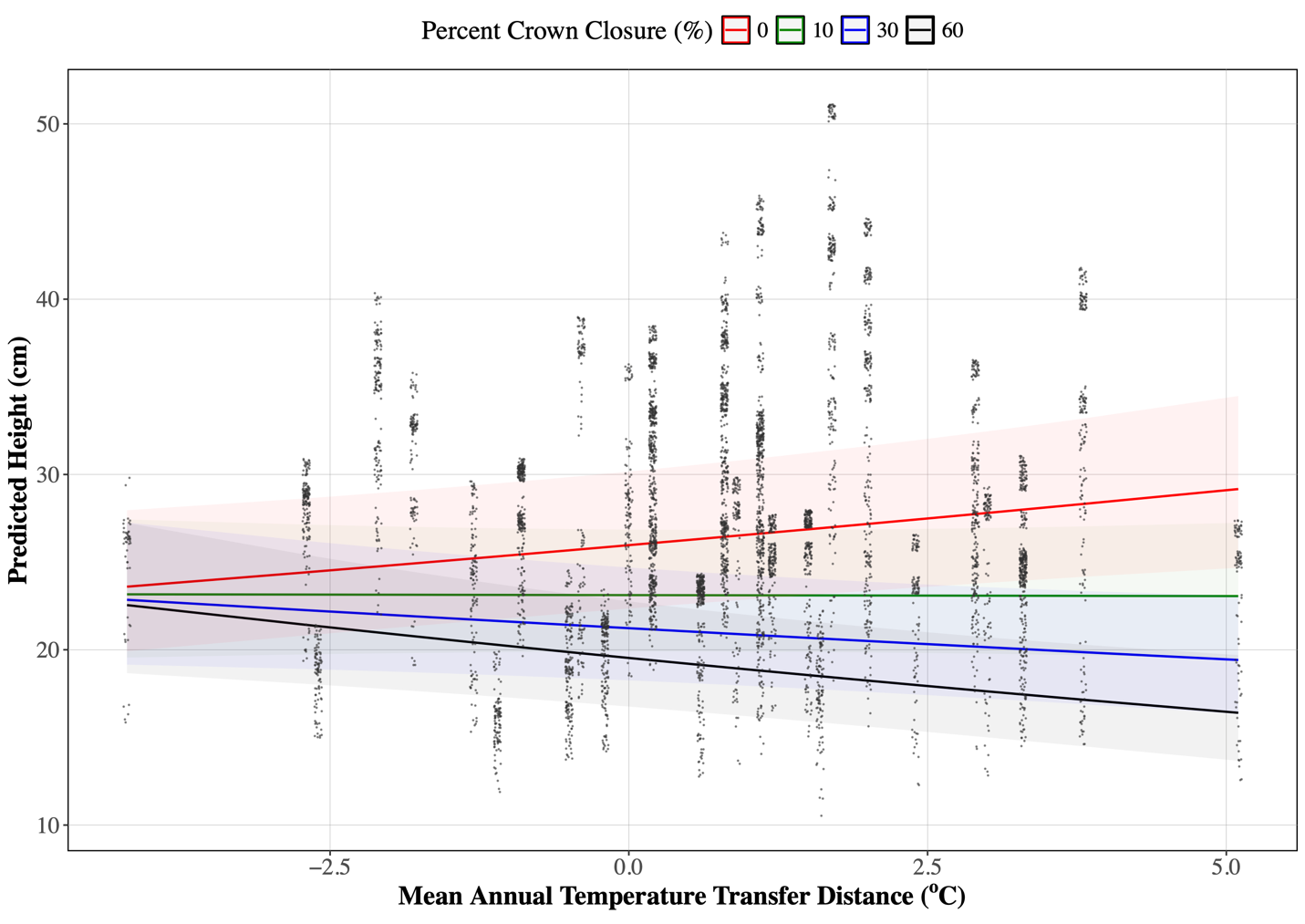
**

**Figure 16.** Predicted height^a^ (cm) against mean annual temperature transfer distance (MAT_td_, ^o^C) for different levels of crown closure (%). Marginal and Conditional R^2^ of 0.055 and 0.379 respectively.

^a^ *ln(height ) ~ Transfer Distance + Crown Closure + Transfer Distance*Crown Closure + (Location/Block/Plot/Splitplot)*
